# The Cyanobacterial Ribosomal-Associated Protein LrtA Is Involved in Post-Stress Survival in *Synechocystis* sp. PCC 6803

**DOI:** 10.1371/journal.pone.0159346

**Published:** 2016-07-21

**Authors:** Carla V. Galmozzi, Francisco J. Florencio, M. Isabel Muro-Pastor

**Affiliations:** Instituto de Bioquímica Vegetal y Fotosíntesis, CSIC-Universidad de Sevilla, Sevilla, Spain; CEA-Saclay, FRANCE

## Abstract

A light-repressed transcript encodes the LrtA protein in cyanobacteria. We show that half-life of *lrtA* transcript from *Synechocystis* sp. PCC 6803 is higher in dark-treated cells as compared to light-grown cells, suggesting post-transcriptional control of *lrtA* expression. The *lrtA* 5´ untranslated leader region is involved in that darkness-dependent regulation. We also found that *Synechocystis* sp. PCC 6803 LrtA is a ribosome-associated protein present in both 30S and 70S ribosomal particles. In order to investigate the function of this protein we have constructed a deletion mutant of the *lrtA* gene. Cells lacking LrtA (∆*lrtA*) had significantly lower amount of 70S particles and a greater amount of 30S and 50S particles, suggesting a role of LrtA in stabilizing 70S particles. *Synechocystis* strains with different amounts of LrtA protein: wild-type, ∆*lrtA*, and LrtAS (overexpressing *lrtA*) showed no differences in their growth rate under standard laboratory conditions. However, a clear LrtA dose-dependent effect was observed in the presence of the antibiotic tylosin, being the LrtAS strains the most sensitive. Similar results were obtained under hyperosmotic stress caused by sorbitol. Conversely, after prolonged periods of starvation, ∆*lrtA* strains were delayed in their growth with respect to the wild-type and the LrtAS strains. A positive role of LrtA protein in post-stress survival is proposed.

## Introduction

Photosynthetic organisms need to harmonize various processes to diurnal changes in light intensity and nutrient availability. Cyanobacteria exhibit a great adaptability to changing environmental conditions through changes in gene expression. Numerous genome expression analyses have been carried out in these organisms, especially in the model *Synechocystis* sp. PCC 6803 (hereafter *Synechocystis*). Most of these studies are related to the transcriptional response to different environmental changes [1]. However, far fewer studies address the translational regulation in cyanobacteria. There is ever-increasing evidence that numerous protein factors interact with the ribosome to regulate protein synthesis and modulate the expression profile of the cell in response to different environmental stresses [2]. In this context, this study aims to advance in the knowledge of LrtA protein as a potential modulator of translation.

The *lrtA* gene was originally identified in *Synechococcus* sp. PCC 7002 as a gene encoding a light-repressed transcript [3]. A deeper study of the regulation and stability of this transcript revealed that its half-life was much higher in darkness than in light [4]. The same study also predicted extensive secondary structure for the 5’ untranslated region (5’ UTR) of *lrtA*, which is also deduced in *Synechocystis lrtA* 5’ UTR. While *lrtA* homologous genes are present in most of the cyanobacterial sequenced genomes the function of LrtA remains unknown. LrtA is related to a family of proteins originally named the sigma-54 modulation proteins, based on the observation that the mutation of the corresponding ORF, downstream of the *Klebsiella pneumoniae rpoN* gene, causes an increase in the expression levels of sigma-54-dependent promoters [5]. Accumulated evidences for several members of this family indicates that these proteins, widespread among bacteria, are ribosome-associated proteins whose function deals with modulation of ribosome activity in order to preserve their integrity and aiding cell survival under stress. Among the most studied members of this family are two *Escherichia coli* proteins: YfiA (pY, RaiA) and YhbH (HPF: hibernation promotion factor). While YfiA is involved in the inactivation of 70S ribosomes, HPF promotes the formation of translationally inactive 100S ribosome particles. These particles result from 70S ribosome dimerization in stationary-phase [6]. Detailed information is available about how these proteins bind to the ribosome and affect protein synthesis [7]. However much less is known about their role *in vivo*. Deletion of *yfiA* or *hpf* genes had no effect on cell growth or cell viability even in the context of stress situations in which these genes are putatively involved [6, 8]. For a broad view of the ribosome regulation in the frame of the bacterial translation stress response, see for example [2, 9, 10].

Phylogenetic analysis revealed that most bacteria have at least one HPF homologue. These homologues have been classified into three types: long HPF, short HPF and YfiA, based on the presence of a conserved domain and additional homologous sequences [11]. According to this classification, cyanobacterial *lrtA* gene might encode a long HPF homologue. Formation of 100S ribosomes is mediated by RMF (ribosome modulation factor) and short HPF in Gammaproteobacteria species, similar to *E*. *coli*, whereas this process is mediated only by long HPF in other bacterial species. The 100S ribosome lacks translational activity, because RMF binds to the 70S ribosome close to the peptidyl transferase center and peptide exit tunnel, and 100S ribosome do not contain tRNA and mRNA [12].

**Fig 1:**
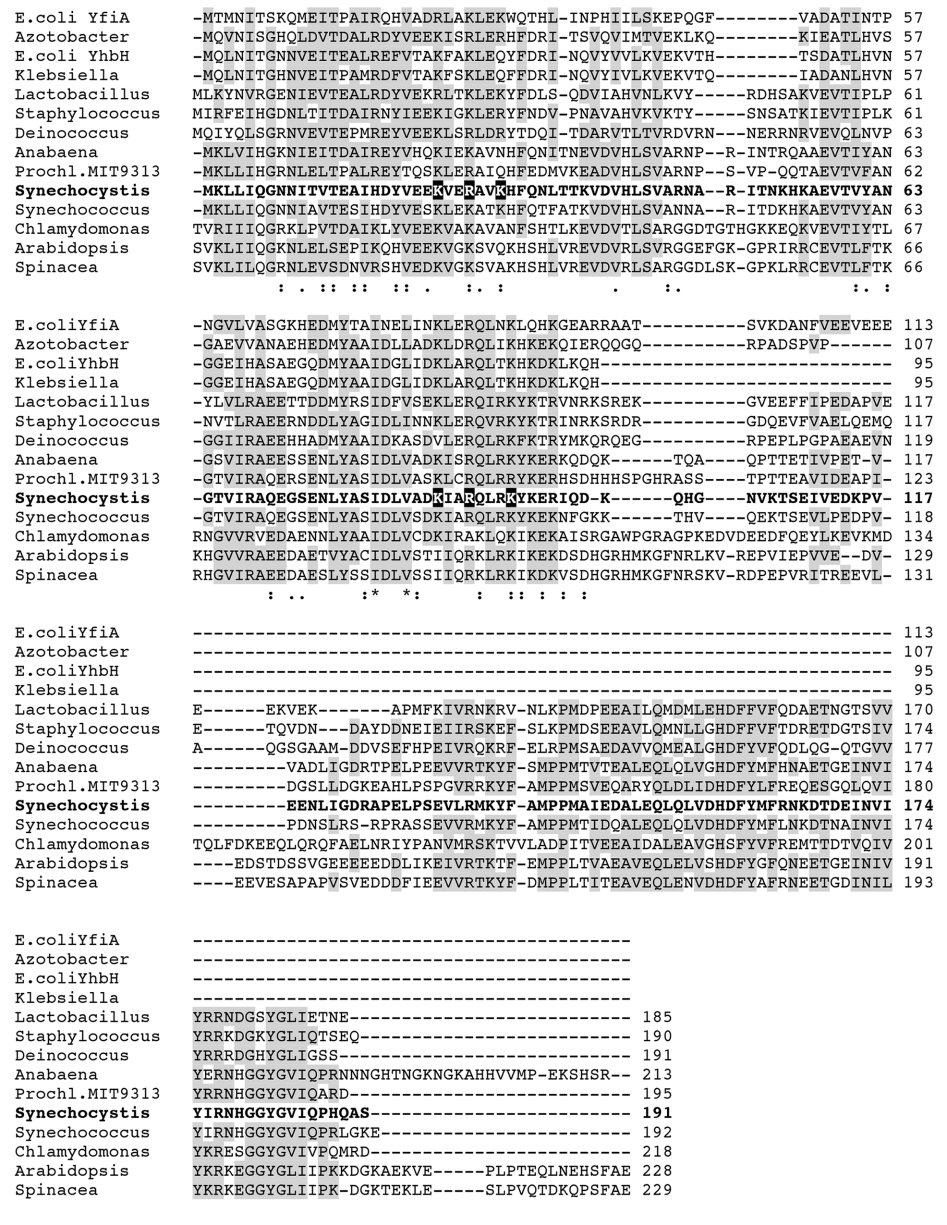
Sequence alignment of several LrtA homologous proteins from bacteria and chloroplasts. E. coli, *Escherichia coli*; Azotobacter, *Azotobacter vinelandii*; Klebsiella, *Klebsiella pneumoniae*, Lactobacillus, *Lactobacillus delbrueckii*; Staphylococcus, *Staphylococcus aureus*; Deinococcus, *Deinococcus radiodurans*; Anabaena, *Anabaena sp*. PCC 7120; Prochl. MIT9313, *Prochlorococcus* MIT 9313; Synechocystis, *Synechocystis sp*. PCC 6803, Synechococcus, *Synechococcus sp*. PCC 7002, Chlamydomonas, *Chlamydomonas reinhardtii*; Arabidopsis, *Arabidopsis thaliana*, Spinacea, *Spinacia oleracea*. In the case of the chloroplastic proteins [*Chlamydomonas reinhardtii* (XP 001697734.1), *Arabidopsis thaliana* (NP 568447.1) and *Spinacia oleracea* (AAA 34039.1)] the chloroplast transit peptide sequence is not shown. Shaded boxes indicate similar amino acids conserved in at least half of the aligned sequences. We considered as similar amino acids the following groups (L,I,V), (K,R), (E,D), (S,T) and (Y,F). The *Synechocystis* sequence is shown in bold and the residues corresponding to the six amino acids involved in ribosome binding in *E*. *coli* YfiA [19] are written in white on black background.

The cyanobacterial *lrtA* gene product also displays sequence similarity to a spinach plastid-specific ribosomal protein (PSRP-1) that is present in the chloroplast stroma either unbound or associated to the 30S ribosomal subunit [13–15]. This observation is consistent with the cyanobacterial origin of the chloroplast [16]. More recent data suggest that PSRP-1 is a functional homologue of the *E*. *coli* pY protein [17, 18]. It stabilizes the ribosome, preventing dissociation, and is recycled by the ribosome-recycling factor (RRF) and translation elongation factor G (EF-G). A sequence alignment of several members of the LrtA protein family is shown in Fig 1.

Our current knowledge about *lrtA* from cyanobacteria is limited. In *Synechocystis*, transcription of *lrtA* is mainly dependent on the group 2 sigma factor SigB, because *lrtA* expression is significantly reduced in a *sigB* knock-out strain. SigB protein level increases 2-fold after a shift from continuous light to darkness [20–22]. In *Synechococcus elongatus* PCC 7942 *lrtA* expression profiles are not dependent on the clock genes (*kai*) since this gene is not significantly altered in the *kaiABC*-null strains [23].

Finally, numerous gene expression and proteomic datasets for *Synechocystis*, under diverse environmental and genetic conditions have been reported in the literature [24]. Although differential accumulation of *lrtA* gene or LrtA protein has been described [25], the function of LrtA remains unknown in cyanobacteria.

In this study we characterize the *lrtA* gene from *Synechocystis*. We show that the cyanobacterial LrtA protein is associated to the 30S ribosomal subunit and that a *lrtA* mutant has a decreased amount of ribosomal 70S particles. A comparative analysis of *lrtA*-null (∆*lrtA*), *lrtA*-overexpressing (LrtAS) and wild-type strains leads us to propose a positive role of LrtA in post-stress survival.

## Materials and Methods

### Bacterial strains, growth conditions and chlorophyll determination

*Synechocystis* was grown photoautotrophically at 30°C in BG11 medium [26] supplemented with 1 g l^-1^ of NaHCO_3_ (BG11C) and bubbled with a continuous stream of 1% (v/v) CO_2_ in air under continuous illumination (50 μmol of photons m^-2^ s^-1^, white light from fluorescent lamps). In cultures with ammonium as nitrogen source 10 mM NH_4_Cl was added to BG11C and the medium was buffered with 20 mM TES (pH 7.5). Dark conditions were obtained by wrapping culture flasks with aluminium foil. For plate cultures, BG11C medium was supplemented with 1% (w/v) agar. Kanamycin was added to a final concentration of 50 μg/ml, spectinomycin and streptomycin were added to a final concentration of 2.5 μg/ml when required.

In the case of antibiotic sensitivity assays tylosin was added in a concentration range between 200 and 300 ng/ml, erythromycin between 10 and 40 ng/ml, tetracycline at 2 and 5 μg/ml, chloramphenicol at 0.25 and 0.5 μg/ml, lincomycin at 5 and 10 ng/ml and puromycin at 5 and 10 μg/ml. *Escherichia coli* DH5α (Bethesda Research Laboratories) grown in Luria Bertani medium was used for plasmid construction and replication. Cultures were supplemented with 100 μg/ml ampicillin or 50 μg/ml kanamycin when required.

Total chlorophyll was measured in methanolic extracts of *Synechocystis* cells [27].

### General DNA manipulations and construction of mutant *Synechocystis* strains

All recombinant DNA experiments were performed according to standard procedures [28]. Southern blotting was done as previously described [28]. To clone the *lrtA* gene, two primers based on the published sequence of the *Synechocystis* genome [29] were synthesized: lrtA1 and lrtA2 (S1 Table). A 1865 bp DNA fragment covering the *lrtA* genomic region (sll0947) was obtained by PCR amplification using these oligonucleotides. This fragment was cloned into pGEM-T to generate pGEM-lrtA.

A *Synechocystis* ∆*lrtA* mutant strain was obtained by replacing the *lrtA* gene with a neomycin phosphotransferase (*npt*) containing cassette (C.K1) [30], which confers kanamycin resistance (Km^r^). The inactivating plasmids pGEM-lrtA::C.K1(+) and pGEM-lrtA::CK1(-) were generated by replacing a 655 bp *Eco*RI-*Bam*HI fragment from pGEM-lrtA by the 1.3 kb *Hinc*II C.K1 cassette, cloned in both orientations. Transformation of *Synechocystis* cells was carried out as previously described [31]. Correct integration of the C.K1 cassette and total segregation of the mutant chromosomes in the ∆*lrtA* (+) and ∆*lrtA* (-) mutant strains was confirmed by Southern blot analysis (S1 Fig).

In order to generate the complemented *Synechocystis* strain (LrtAC), a wild type *lrtA* gene was introduced in the ∆*lrtA* mutant strain by transformation. A 1206 bp DNA fragment including *lrtA* promoter, 5’-UTR and ORF was amplified by PCR from *Synechocystis* genomic DNA, using the oligonucleotides lrtAKpnI and lrtASmaI (S1 Table). This fragment was cloned in a plasmid containing a 2 kb region of the non-essential *nrsBACD* operon, used as a platform for integration of constructs [32]. A streptomycin/spectinomycin resistance cassette from pHP45Ω [33] was placed downstream of the *lrtA locus* (S1 Fig). The resulting plasmid was used to transform ∆*lrtA Synechocystis* cells and the integration of *lrtA* gene into the *nrsBACD* operon by homologous recombination was confirmed by Southern blot analysis.

In order to overexpress *lrtA* under control of the P_trc_ promoter, a 591 bp DNA fragment containing *lrtA* ORF was amplified by PCR from *Synechocystis* genomic DNA, using the oligonucleotides lrtABspHI and lrtASmaI.2 (S1 Table). This fragment was cloned into pTrc99A *Nco*I-*Sma*I digested [34]. An *EcoR*V-*Sma*I fragment from the resulting plasmid, containing the P_trc_ promoter, a ribosome binding site from pTrc99A and the *lrtA* ORF, was cloned in a plasmid harbouring a 2 kb region of the non-essential *nrsBACD* operon [32]. A streptomycin/spectinomycin resistance cassette from pHP45Ω [33] was placed downstream of the *lrtA* ORF. The resulting plasmid was used to transform wild-type or ∆*lrtA Synechocystis* cells, producing LrtAS_WT and LrtAS_∆*lrtA*, respectively.

### Primer extension analysis

Primer extension analysis was carried out using oligonucleotide lrtAR4 (S1 Table) as previously described [35].

### RNA isolation and Northern blot hybridization

Total RNA was isolated from 25 ml samples of *Synechocystis* cultures at the early-exponential phase (3–4 μg chlorophyll/ml), except for experiments involving different growth phases. RNA extraction was performed by vortexing cells in the presence of phenol:chloroform and acid washed baked glass beads (0.25–0.3 mm diameter; Braun, Melsungen, Germany) as previously described [36]. RNA blotting to nylon membranes (Hybond N-plus; Amersham), prehybridization, hybridization and washes were in accordance with Amersham instruction manuals. A 655 bp *Eco*RI-*Bam*HI fragment from plasmid pGEM-lrtA was used as *lrtA* probe for Northern blotting experiments. In order to quantify signals, in all the cases the filters were reprobed with a *Hin*dIII-*Bam*HI 580 bp probe from plasmid pAV1100 that contains the constitutively expressed *rnpB* gene from *Synechocystis* [37]. To determine cpm of radioactive areas in Northern blot hybridizations either an InstantImager Electronic Autoradiography apparatus (Packard Instrument Company, Meriden, CT) or a Cyclone Phosphor System (Packard) were used.

### LrtA expression and purification

The pGEM-lrtA-his plasmid was created by replacing the stop codon of the *lrtA* gene of pGEM-lrtA by 6 histidine codons followed by a new stop codon. An *Nde*I-*Bam*HI fragment from pGEM-lrtA-his plasmid encompassing the *lrtA-his* gene was cloned into *Nde*I-*Bam*HI digested pET3a plasmid (Novagen, La Jolla, CA) to generate pET3a-lrtA-his. Exponentially growing *E*. *coli* BL21 cells transformed with pET3a-lrtA-his were treated with 0.5 mM of isopropyl ß-D-1-thiogalactopyranoside for 4 h. For purification of LrtA-His_6_ cells were collected, resuspended in buffer A (20 mM sodium phosphate, pH 7.4, 0,5 M NaCl) with 1 mM phenylmethylsulfonyl fluoride and disrupted by sonication. The lysate was centrifuged at 18,000 x *g* for 10 min. LrtA-His_6_ from the supernatant was purified by Ni-affinity chromatography using His-Bind resin matrix (Novagen) and following the manufacturer’s instructions.

### Anti-LrtA antibody production and Western Blotting

Anti-LrtA antiserum was obtained according to standard immunization protocols by injecting one milligram of purified LrtA-His_6_ protein in rabbits. Antibodies against *E*. *coli* S12 and L13 proteins were kindly provided by Richard Brimacombe.

For Western blot analysis proteins were fractionated on 12% SDS-PAGE according to the method of Laemmli [38] and immunoblotted with anti-LrtA (1:4000), anti-S12 (1:2000) or anti-L13 (1:2000). The ECL Plus immunoblotting system (Amersham) was used to detect the different antigens with anti-rabbit (1:12,000) or anti-sheep (1:5,000) secondary antibodies conjugated to horseradish peroxidase.

### Preparation of crude extracts from *Synechocystis* cells

To analyse the abundance of LrtA in cells of *Synechocystis* grown under different conditions, crude extracts were prepared using glass beads as previously described [39] in 50 mM Hepes-NaOH buffer, pH 7.0, 50 mM KCl. Equal volumes (typically 10 μl) of the processed samples were loaded on SDS-PAGE.

### Isolation of ribosomal particles

For ribosome isolation 3 l of *Synechocystis* cultures were collected by centrifugation at 10.000 x g. Cells were mixed in a mortar with double weight of alumina, broken by grinding and resuspended in buffer A (20 mM Tris-HCl pH 8.0, 20 mM NH_4_Cl, 5 mM ß-mercaptoethanol) supplemented with 1 or 10 mM magnesium acetate, in order to dissociate or not the 30S and 50S ribosomal subunits. The crude extract was centrifuged at 10.000 x *g* for 10 min to eliminate alumina and unbroken cells. The supernatant was then centrifuged at 30.000 x *g* for 30 min and the supernatant was collected (S30 fraction). 200–300 μl of S30 fraction (approximately 1 mg of protein) was layered onto 10–30% continuous sucrose density gradient in buffer A with the appropriated magnesium acetate concentration and centrifuged at 2°C for 8 h at 25.000 rpm, using a Beckman SW41 Ti rotor. Gradients were analyzed with an ISCO UA-6 detector with continuous monitoring at 254 nm. 0.5 ml fractions were collected and total protein was precipitated with 10% trichloroacetic acid.

## Results

### *Synechocystis lrtA* transcript analysis under light-dark transitions

**Fig 2:**
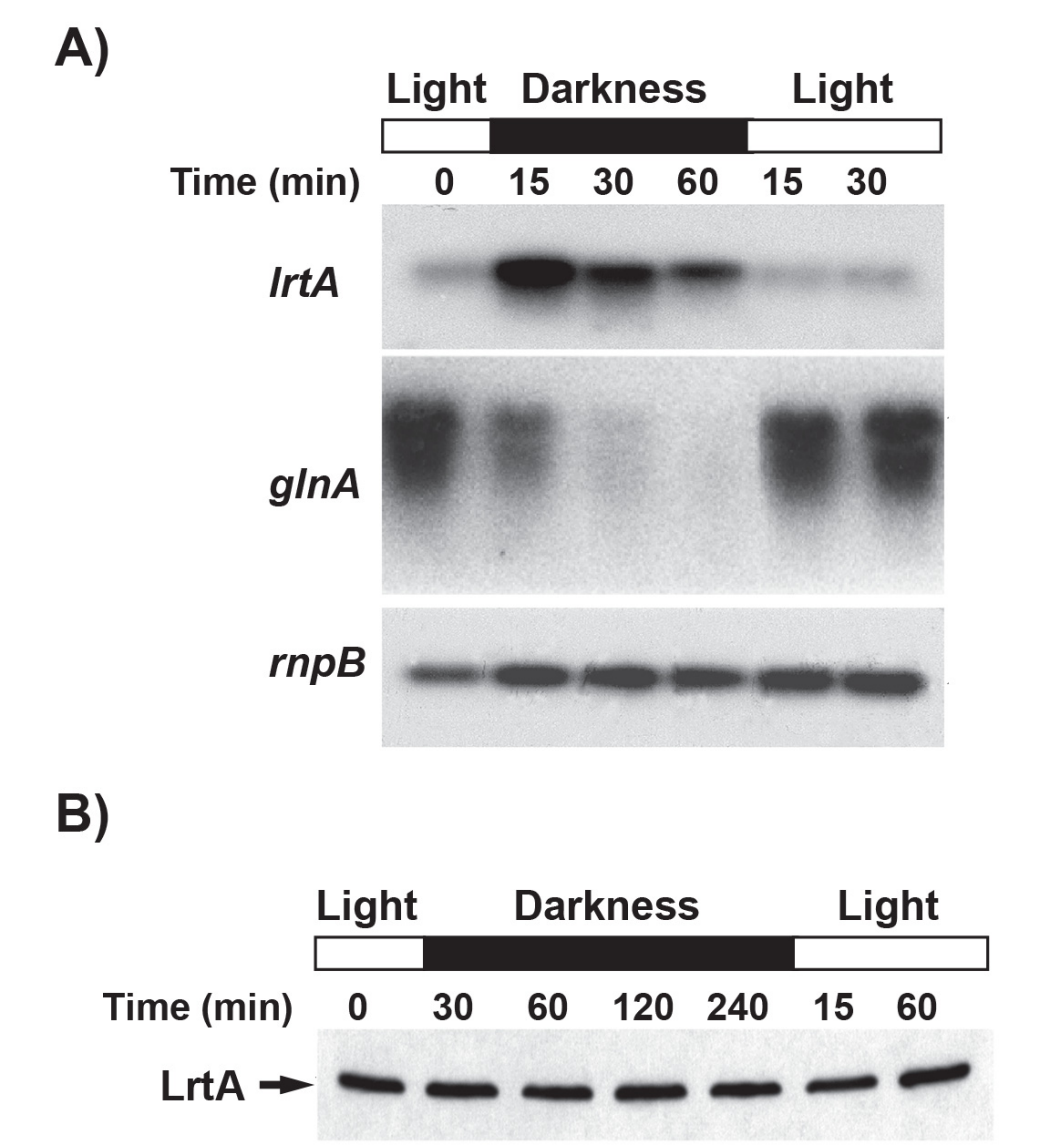
Darkness-dependent regulation of *lrtA* mRNA and protein levels. *A*, Total RNA was isolated from early-log-phase *Synechocystis* cells growing under normal illumination conditions (Light), after being subjected to darkness for 15, 30 or 60 min, and after re-illumination of the culture for 15 or 30 min. 15 μg of total RNA was loaded per lane. Levels of *lrtA* and *glnA* mRNA were determined by Northern blotting. All the filters were stripped and re-hybridized with a *rnpB* gene probe as loading control. The data shown are representative of four independent Northern-blot experiments showing similar results. *B*, Level of LrtA protein was determined by Western blotting using anti-LrtA serum. 6 μg of total protein was loaded per lane. The data shown are representative of three independent Western-blot experiments showing similar results.

The analysis of the *Synechocystis* genome showed the existence of an open reading frame (sll0947) product that displayed strong similarity to the previously identified *Synechococcus* sp. PCC 7002 LrtA protein (Fig 1) [3]. We analyzed the transcript level of the *Synechocystis lrtA* gene under light-dark transitions and found that *lrtA* mRNA increased around 7 times 15 minutes after transferring exponentially growing cells (2–3 μg chlorophyll/ml of culture) to the dark (Fig 2A). The *lrtA* mRNA level quickly decreased upon re-illumination of the culture. Transcription of many cyanobacterial genes shows an inverse behaviour upon a light-dark shift, reflecting a decreased metabolism of these photosynthetic organisms under darkness [40]. Therefore, as a control, Fig 2A also shows that under the same conditions the *glnA* (the gene encoding glutamine synthetase I) mRNA followed an inverse pattern to *lrtA*. The darkness-dependent up-regulation of the *lrtA* mRNA was found to be transitory. The maximal level of *lrtA* mRNA was observed within 15 min of darkness, and thereafter transcript level decreased slowly, reaching level similar to those present under continuous illumination after 3–4 h (Fig 2A and S2 Fig). In contrast to the *lrtA* mRNA, the level of the LrtA protein did not change in response to light-dark-light switches as evidenced by Western blotting experiments using an anti-LrtA polyclonal serum (Fig 2B).

**Fig 3:**
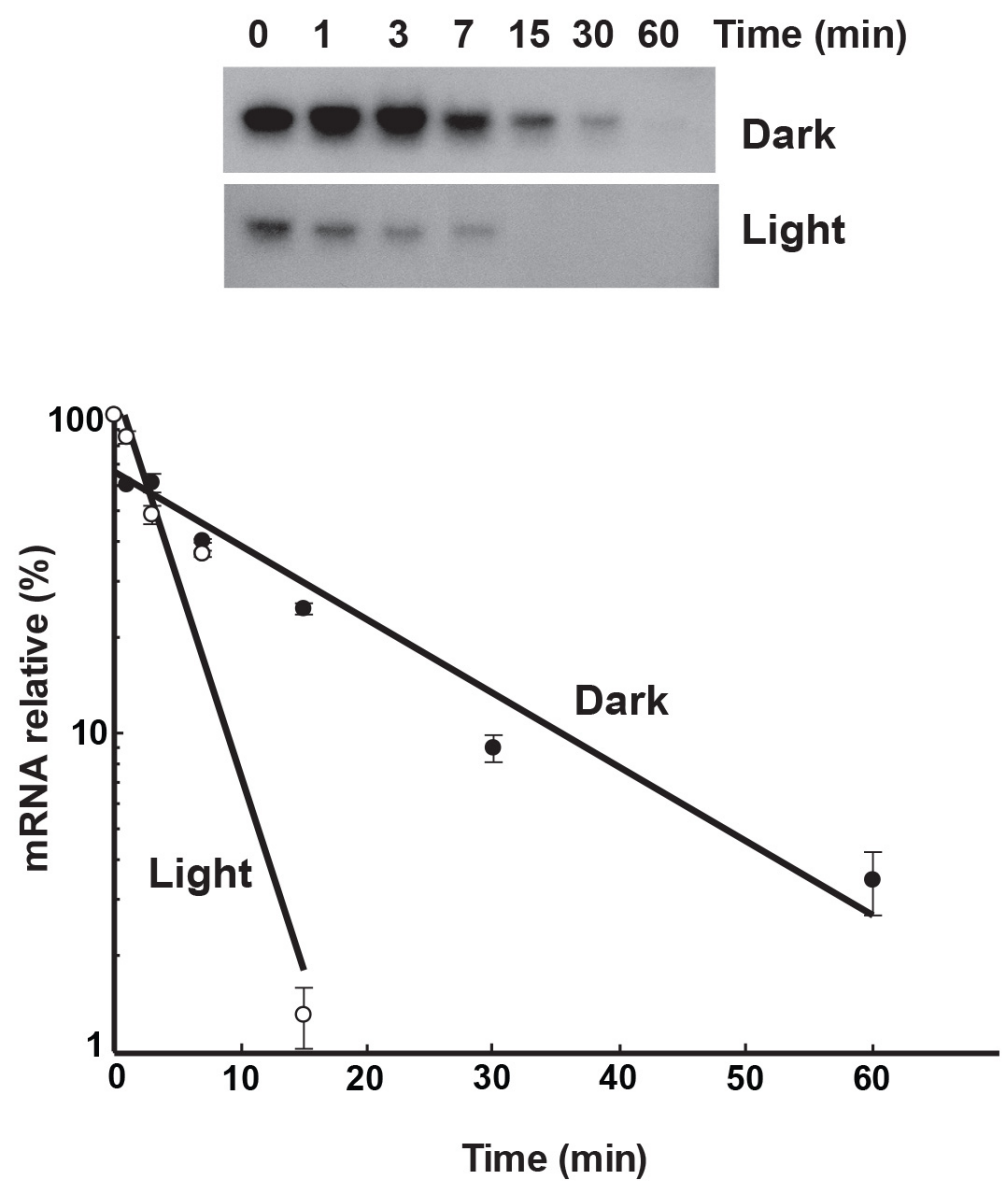
Dark-increased stability of *lrtA* transcript. Rifampicin (400 μg/ml) was added to light (Light) growing *Synechocystis* cells or to 15 min dark-treated cells (Dark). Samples were taken, under the same conditions, at the indicated times and total RNA was isolated. 15 μg of total RNA was loaded per lane. Levels of *lrtA* mRNA were determined by Northern blotting. Radioactive signals intensity were normalized with respect to the *rnpB* RNA level, and plotted against time. Values are the average of two independent experiments.

The effect of darkness on the *lrtA* transcript stability was further investigated. To that end, transcription was blocked by addition of rifampicin to *Synechocystis* cells in the light or in the dark. Samples were taken at different times and transcript levels were determined by Northern blotting. In the light, the *lrtA* transcript level decreased with a half-life of 3.2 ± 0.1 min. However, the *lrtA* transcript from dark-treated cells showed a longer half-life (5.3 ± 0.25 min) (Fig 3). These data suggest that a post-transcriptional mechanism is responsible for the darkness-dependent regulation of the *lrtA* gene.

### Expression of the *lrtA* gene is controlled by the growth phase

**Fig 4:**
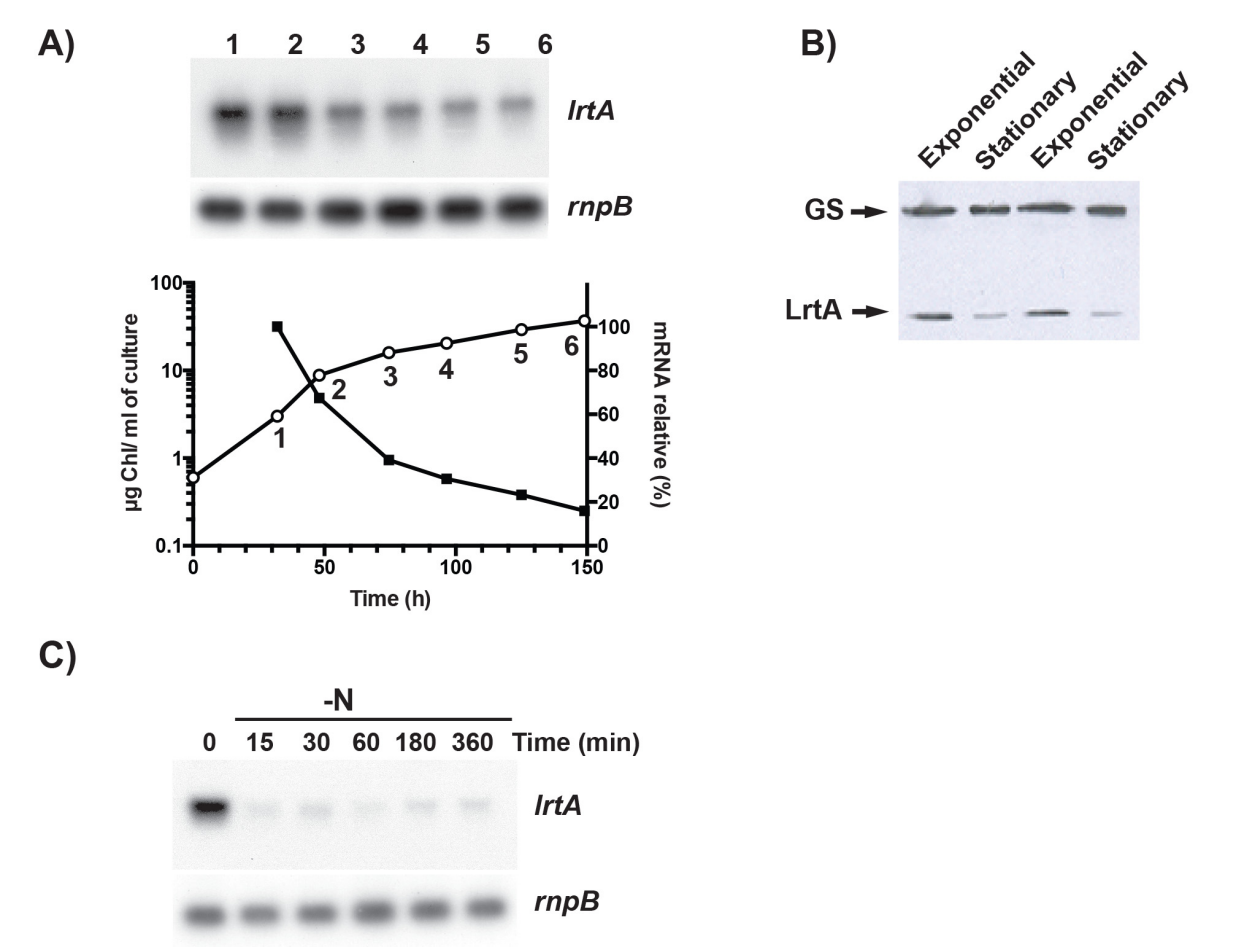
*lrtA* mRNA and protein levels as a function of cell growth. *A*, Aliquots of *Synechocystis* cells growing in BG11C medium were taken at different cell densities. Chlorophyll was measured in methanolic extracts (open circles). Total RNA was isolated from each sample (numbers in the plot), 15 μg of total RNA was loaded per lane and levels of *lrtA* mRNA were determined by Northern blotting. Radiactive signals were quantified, values were normalized with respect to the *rnpB* RNA level (mRNA relative) and plotted (closed squares). Data are expressed in percentage of the maximum value obtained in the exponential growth phase. The data shown are representative of three independent Northern-blot experiments showing similar results. *B*, LrtA level is affected by the growth phase. Total protein was isolated from exponential-growth-phase *Synechocystis* cells (2.2 μg Chl/ml of culture) or late linear-growth-phase cells (>20 μg Chl/ml of culture). 5 μg of total protein was loaded per lane. Level of LrtA protein was determined by Western blotting using anti-LrtA serum. As a control for protein loading, membranes were also incubated with anti-GSI. Glutamine synthetase I (GSI) is constitutively expressed, independently of the growth phase in *Synechocystis* cells. The data shown are representative of three independent Western-blot experiments showing similar results. *C*, Early exponentially growing *Synechocystis* cells that used ammonium as nitrogen source (time 0) were centrifuged, washed and resuspended in BG11C medium lacking any nitrogen source (BG11_0_C). Aliquots of culture were taken at the indicated time for total RNA isolation and levels of *lrtA* mRNA were determined by Northern blotting. 15 μg of total RNA was loaded per lane. The data shown are representative of three independent Northern-blot experiments showing similar results.

*E*. *coli* proteins related to LrtA, YfiA and YhbH, have been shown to be up-regulated during stationary phase [41]. In order to investigate whether *Synechocystis lrtA* is controlled in a similar way, the level of *lrtA* transcript was determined by Northern blot at different time points along the growth curve. As shown in Fig 4A, the level of the *lrtA* transcript was maximal in the early exponential growth phase, thereafter decreasing progressively as the cell density increased. The level of LrtA protein was also lower when cultures were reaching stationary phase (Fig 4B).

Stationary growth phase is reached by a deficiency in one or more macronutrients. We investigated whether nitrogen starvation also promotes a decrease in the level of *lrtA* mRNA. For that, exponentially growing *Synechocystis* cells using ammonium as nitrogen source were transferred to a nitrogen-free medium. Fig 4C shows that *lrtA* expression is strongly down regulated by nitrogen deficiency.

### The *lrtA* 5´ untranslated leader region is involved in darkness-dependent regulation

Determination of the *Synechocystis lrtA* transcription start point by RNA primer extension analysis demonstrated the presence of a long untranslated leader region in the *lrtA* mRNA. Thus, only one transcription start site was found 312 bp upstream of the *lrtA* ATG start codon (S3 Fig). This result agrees with the previously described transcription initiation for the *lrtA* gene by both primer extension [22] and transcriptomic [42].

To characterize the function of the *lrtA* gene, a strain lacking this gene was generated by replacing the complete open reading frame by a kanamycin resistance cassette (see M&M for details). Complete segregation of the mutation and absence of *lrtA* transcript were confirmed by Southern blotting and Northern blotting, respectively. The absence of the LrtA protein was also confirmed by Western blotting experiments (S1 Fig). The *∆lrtA* strain was viable, indicating that *lrtA* is a dispensable gene in *Synechocystis*.

**Fig 5:**
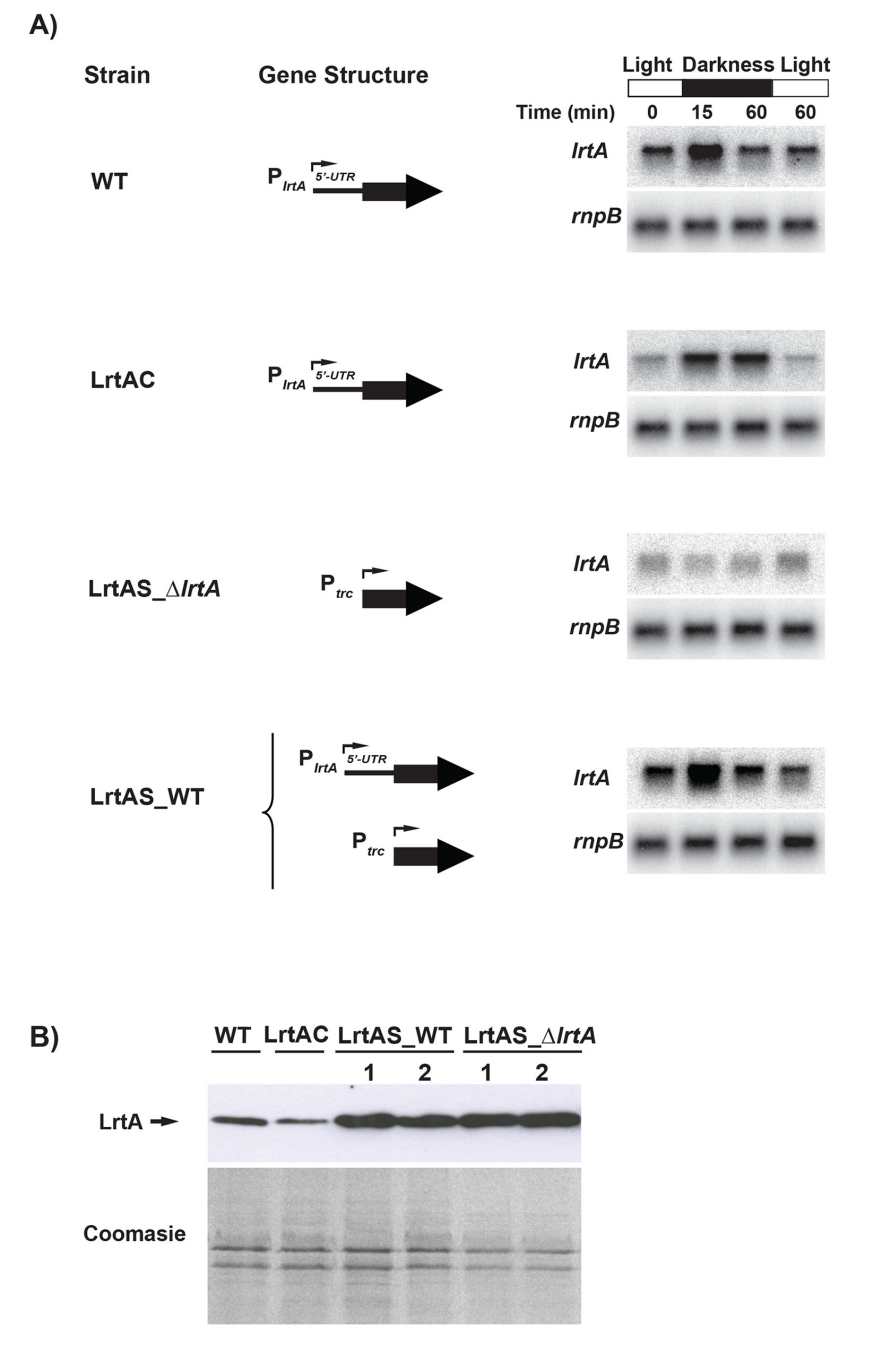
*lrtA* mRNA and protein levels in different *Synechocystis* strains. In the LrtAC strain a copy of the *lrtA locus*, including its promoter and 5’UTR region, was introduced in the dispensable *nrsBACD locus* of *Synechocystis*. LrtAS_WT and LrtAS_*∆lrtA* strains harbour the *lrtA* ORF (without the leader region) fused to the P_trc_ promoter in the *nrsBACD locus*, in a wild type and *lrtA*-deleted background, respectively. *A*, Total RNA was isolated from early-log-phase *Synechocystis* cells of the different strains, growing under normal illumination conditions (Light), after transferring of the cultures to the dark for 15 or 60 min (Darkness), or after re-illumination of the cultures for 60 min. 8 μg of total RNA was loaded per lane and levels of *lrtA* mRNA were determined by Northern blotting. All the filters were stripped and re-hybridized with a *rnpB* gene probe. The data shown are representative of three independent Northern-blot experiments showing similar results. *B*, Level of LrtA protein, under normal illumination conditions, was determined by Western blotting using anti-LrtA serum. 6 μg of total protein was loaded per lane. As a loading control a Coomassie-stained SDS-PAGE gel of the samples used for Western blot is shown. The data shown are representative of three independent Western-blot experiments showing similar results.

To investigate the position of putative cis-regulatory elements responsible for the darkness-dependent regulation of the *lrtA* gene we generated a construct were the *lrtA* ORF (without the leader region) was fused to the P_trc_ promoter, which was reported to act as a constitutive promoter in *Synechocystis* [43]. This *lrtA* version was introduced in the dispensable *nrsBACD locus* of *Synechocystis* [32], either in the wild type strain or in the *lrtA*-deleted strain, obtaining strains LrtAS_WT and LrtAS_*∆lrtA*, respectively. As a control, a wild type version of the *lrtA locus*, including its promoter and 5’UTR region, was introduced in the same *nrsBACD locus* of the *lrtA*-deleted strain, obtaining the complemented strain LrtAC (S1 Fig). *lrtA* transcript in each strain was analysed by Northern blot (Fig 5A). The results with the LrtAS_*∆lrtA* strain showed that accumulation of the *lrtA* transcript in this strain is lower compared to the wild type strain. In addition, the pattern of expression is opposite to the one observed in wild type cells, namely, the *lrtA* transcript does not increase under dark conditions. On the other hand, the level of *lrtA* transcript in the LrtAS_WT is indistinguishable from that of the wild type under all conditions. Therefore our data suggest that the darkness-dependent increase in the abundance of the *lrtA* transcript depends on the leader region. Fig 5B shows the analysis by Western blot of the LrtA protein in the different strains under light conditions. Surprisingly, both LrtAS_WT and LrtAS_*∆lrtA* strains show a higher LrtA protein abundance compared to the wild type strain. This result indicates that the leader-less transcript synthesized from the P_trc_ promoter must be translated efficiently. Regarding the complemented strain (LrtAC), both the level of the *lrtA* transcript and LrtA protein are slightly lower with respect to the wild type strain but the darkness-dependent regulation of the transcript is not altered. Taken together, these results suggest that the *lrtA* leader region stabilizes the transcript but hampers its translation. As mentioned in the introduction, extensive secondary structure can be predicted for the 5’UTR leader region of *lrtA* gene. One of these predictions is presented as supplemental material (S4 Fig).

### LrtA is associated to ribosomal particles

**Fig 6:**
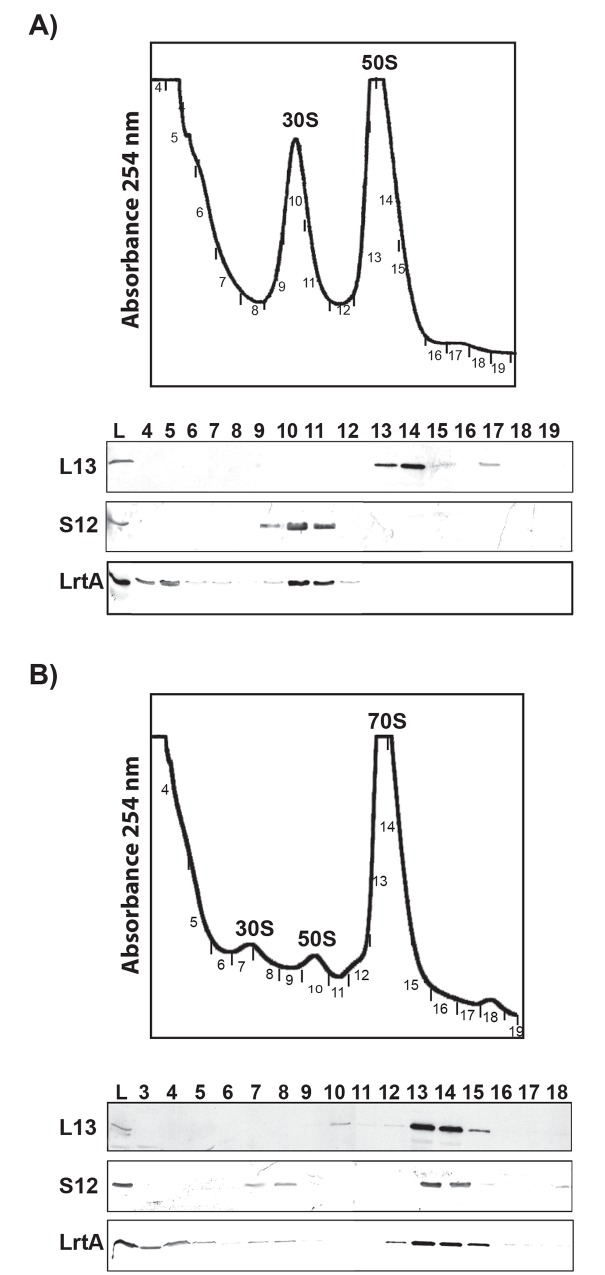
Association of LrtA with ribosomal subunits. The *Synechocystis* S30 supernatant was fractionated on a 10–20% sucrose density gradient in the presence of 1 mM (A) or 10 mM (B) magnesium acetate. Top: sedimentation profiles showing the free subunits and 70S ribosomes. Bottom: equal volumes of each fraction were subjected to Western blotting using anti-LrtA, anti-L13 and anti-S12 antibodies. The data shown are representative of three independent sedimentation profiles and Western-blot analysis showing similar results.

**Fig 7:**
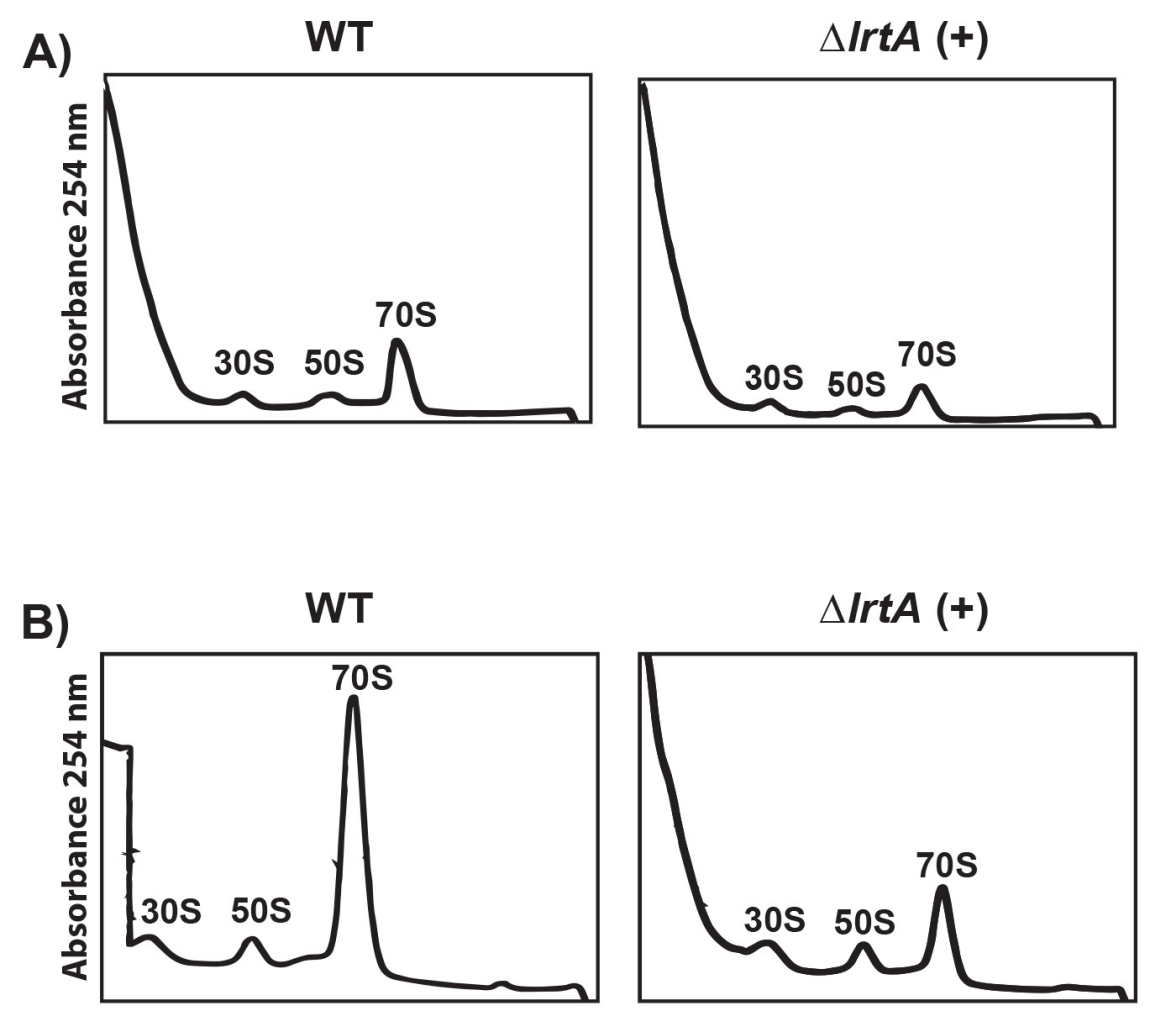
Absence of LrtA diminishes 70S particles amount. *Synechocystis ∆lrtA* and wild type strain S30 supernatants were fractionated on 10–20% sucrose density gradients in the presence of 10 mM magnesium acetate. Sedimentation profiles, showing the free subunits and 70S ribosomes, were obtained from two different stages of the cultures for each strain: *A*, about 2 μg Chl/ml and *B*, about 15 μg Chl/ml. ∆*lrtA*(+) refers to the orientation of the antibiotic resistance cassette in the same direction respect to the *lrtA* gene. The data shown are representative of three independent sedimentation profiles experiments showing similar results.

Based on *lrtA* sequence homologies, we investigated whether LrtA is a ribosomal protein in cyanobacteria. For this purpose, extracts from *Synechocystis* cells were fractionated by sucrose gradient centrifugation using buffers with a low or a high Mg^2+^ concentration. Proteins from individual fractions were separated by SDS-PAGE and LrtA was detected by Western blotting. Antibodies directed against L13 and S12 were also used as markers for the 50S and 30S subunits, respectively. As shown in Fig 6, LrtA was found to be associated to both the 30S and the 70S ribosomal particles. A significant amount of LrtA was also detected as a free form that does not seem to be associated to ribosomal particles. On the other hand, negligible amount of 100S ribosomal particles could be detected in our ribosomal profiles regardless of the growth phase (Figs 6 and 7).

### The lack of LrtA affects the abundance of 70S particles

Considering the observed LrtA association with 30S and 70S ribosomal particles, we investigated whether the absence of LrtA may have any effect on the ribosomal profiles in *Synechocystis*. Extracts from *Synechocystis ∆lrtA* and wild type strain cells were fractionated by sucrose gradient centrifugation using buffers with 10 mM Mg^2+^ concentration. As shown in Fig 7 *∆lrtA* strain displayed significantly lower amount of 70S particles and a higher amount of 30S and 50S particles could be also detected in the ribosome profiles from the mutant strain. This effect was observed independently of the grow phase of the cells analyzed. These results suggest a role of LrtA in stabilizing 70S particles.

### The absence of LrtA is an advantage under some stress conditions

**Fig 8:**
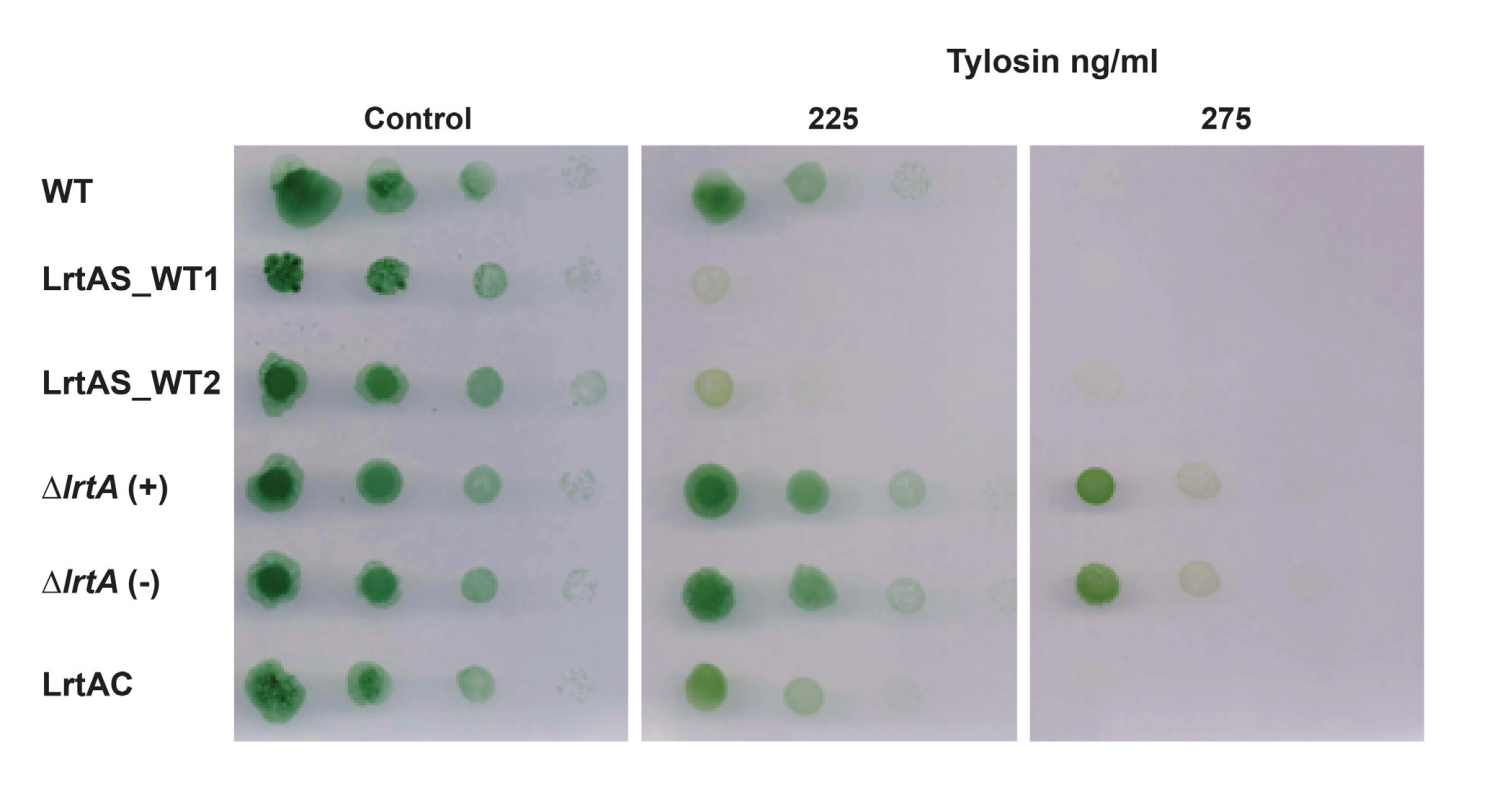
Effect of tylosin on the growth of *Synechocystis* strains with different LrtA amounts. Ten-fold serial dilutions of each *Synechocystis* culture were spotted on BG11C medium plates (control) or in BG11C supplemented with the indicated concentration of antibiotic and photographed after 10 days of growth. ∆*lrtA*(+) and ∆*lrtA*(-) refer to the orientation of the antibiotic resistance cassette in the same or opposite direction respect to the *lrtA* gene, respectively. The data shown are representative of three independent experiments showing similar results.

Some LrtA homologues have been related to bacterial stress response [2]. We therefore analysed the phenotype of strains lacking LrtA under certain stress conditions. Since the ribosome is one of the major targets for antibiotics, we measured sensitivity of ∆*lrtA* cells to low concentrations of antibiotics that inhibit protein synthesis. ∆*lrtA* showed similar sensitivity than wild type cells to tetracycline, chloramphenicol, lincomycin, and puromycin (S5B Fig). However, ∆*lrtA* cells were more resistant to tylosin and erythromycin (S5A Fig) than wild-type cells. Wild type, ∆*lrtA*, the complemented strain LrtAC and the overexpressing strains LrtAS were tested for their sensitivity to tylosin. A clear LrtA dose-dependent effect was observed, being the LrtA-overexpressing cells the most sensitive to tylosin (Fig 8).

**Fig 9:**
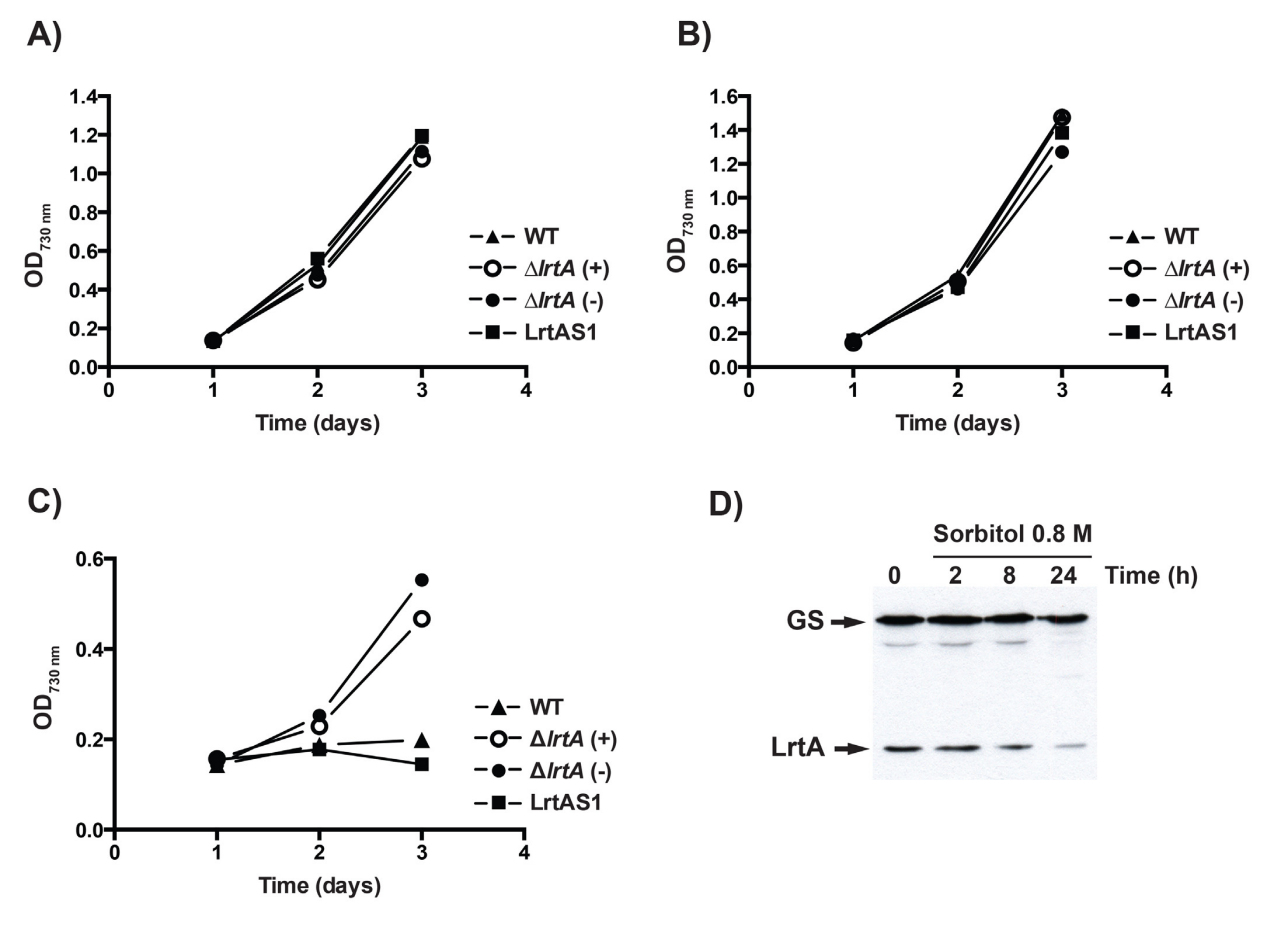
Effect of sorbitol and NaCl on the growth of *Synechocystis* strains with different LrtA amounts. Growth curves of the indicated strains under standard conditions in BG11C medium (*A*), in BG11C supplemented with 0.7 M NaCl (*B*) or in BG11C with 0.5 M sorbitol (*C*). ∆*lrtA*(+) and ∆*lrtA*(-) refer to the orientation of the antibiotic resistance cassette in the same or opposite direction respect to the *lrtA* gene, respectively. Each growth curve represents the mean of three independent experiments showing similar results. *D*, LrtA level is affected by the presence of sorbitol. Total protein was isolated from exponentially growing *Synechocystis* cells incubated with 0.8 M sorbitol for up to twenty-four hours. 5 μg of total protein was loaded per lane. Level of LrtA protein was determined, at different times after sorbitol addition, by Western blotting using anti-LrtA serum. As a control for protein loading, membranes were incubated also with anti-GSI. The data shown are representative of three independent Western-blot experiments showing similar results.

It has been described that salt stress and hyperosmotic stress have different effects on gene expression and cytoplasmic volume in *Synechocystis* cells [44]. On the other hand, SigB protein, involved in transcribing *lrtA* gene, regulates salt acclimation responses in *Synechocystis* [45]. We have studied the effect of these stress conditions on the growth rate of *Synechocystis* cells with different levels of LrtA protein. While the salt stress (0.7 M NaCl) appears to have no significant effect on the growth of different strains, hyperosmotic stress (0.5 M sorbitol) differentially affects wild type, LrtAS and ∆*lrtA* strains. Remarkably ∆*lrtA* cells are able to grow under hyperosmotic stress conditions (Fig 9). We also tested the effect of high concentration sorbitol (0.8 M) treatment on LrtA protein abundance in wild-type cells. As shown in Fig 9D, LrtA protein amount decreases after sorbitol addition. This result is in agreement with the pronounced growth observed in ∆*lrtA* cells under osmotic stress (Fig 9C).

### LrtA affects post-stress survival of *Synechocystis* cells

**Fig 10:**
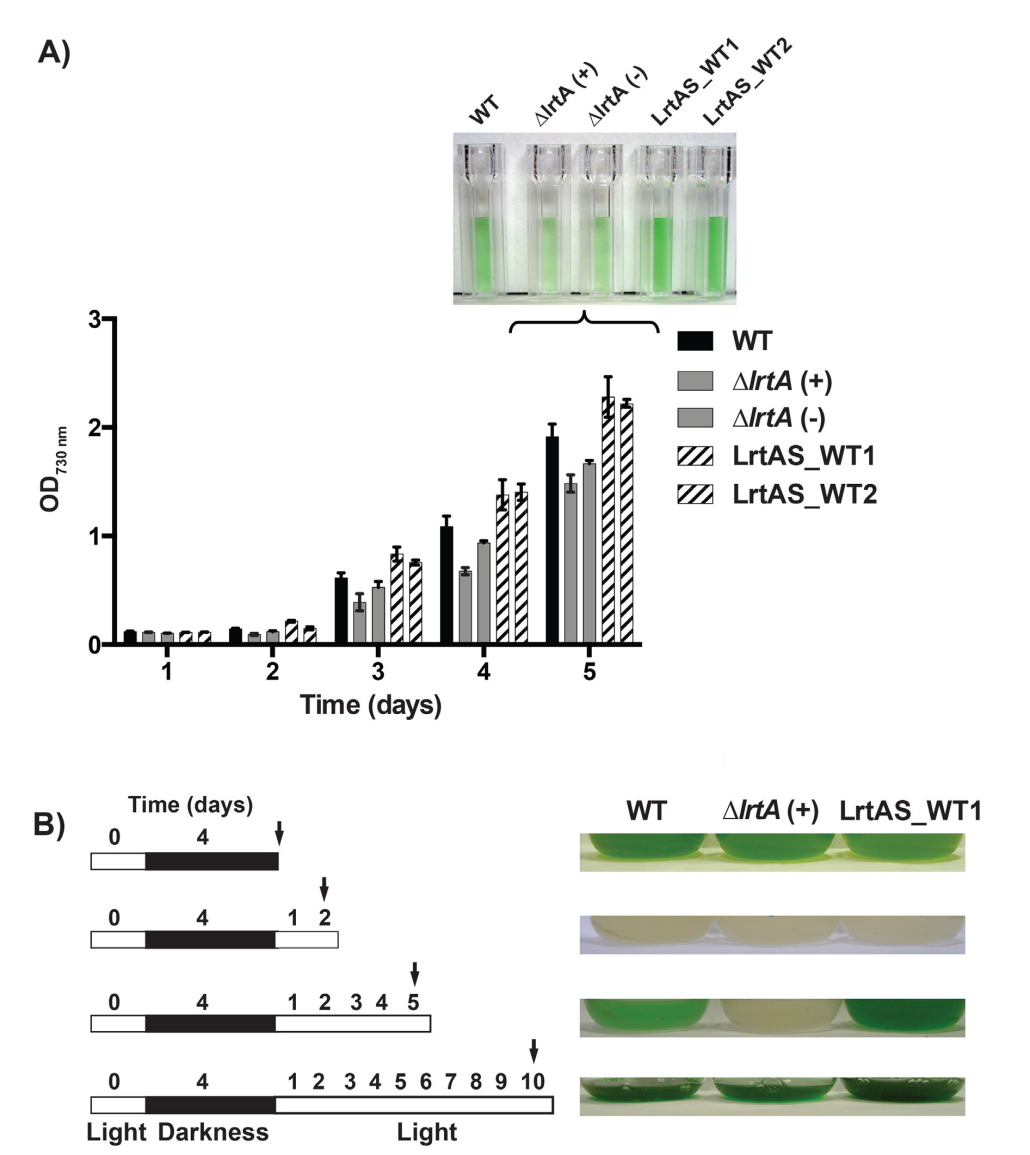
Growth of *Synechocystis* strains with different amounts of LrtA, in refreshing conditions after stationary phase. *A*, Cells of the wild-type and mutant strains were cultivated photoautotrophically (50 μmol photons m^-2^ s^-1^, 1% (v/v) CO_2_ in air) during three weeks. These cells were used to inoculate fresh medium at 0.1 OD_730_ (day 1). Growth was followed for 5 days and at that time aliquots of the cultures were photographed. The data represent average values of three independent experiments. *B*, Growth after darkness treatment. At day “0” exponentially growing cells under standard illumination conditions (light) were transferred to darkness during four days and then re-illuminated. Arrows indicate the day on which the cultures were photographed. ∆*lrtA*(+) and ∆*lrtA*(-) refer to the orientation of the antibiotic resistance cassette in the same or opposite direction respect to the *lrtA* gene, respectively.

*Synechocystis* strains with different LrtA protein levels (wild-type, ∆*lrtA*, and LrtAS) showed similar growth rates under standard conditions (Fig 9A). To further investigate the physiological role of LrtA, these strains were subjected to different treatments leading to slowing or stopping the growth by the lack of light or nitrogen. Following the starvation period, growth of the different strains was evaluated under favourable conditions. In the first type of experiments, cells were cultured photoautotrophically (50 μmol photons m^-2^ s^-1^, 1% (v/v) CO_2_ in air) for three weeks, reaching the late stationary phase and high cell density. Then these cells were used to inoculate fresh medium at low optical density (OD_750_ = 0.1) and evolution of the cultures was monitored for five days. As shown in Fig 10A, there was a positive correlation between cell growth and the amount of LrtA protein present in each strain. LrtA lacking cell are delayed in their growth with respect to the wild-type and the overexpressing strains. The same results were obtained with nitrogen starved cells used to restart cultures in nitrate-containing BG11C medium (S6 Fig). In the second type of experiments, early-log phase cells of the different strains growing under standard illumination (50 μmol photons m^-2^ s^-1^) were transferred to darkness for four days. After this period the cultures were re-illuminated and their evolution was followed visually. As shown in Fig 10B, re-illumination after the four-days darkness period, provoked a drastic lost of pigmentation that was clearly appreciated two days following re-illumination. This effect was identical in all the strains. However, cells with different LrtA amount restarted growth differentially. LrtA-overexpressing cells recovered faster than wild-type cells whereas LrtA-lacking cells exhibited a remarkable slow recovery.

## Discussion

Adaptation to changes in the environment such as cold-shock, heat-shock, nutrient deprivation, and stationary phase, is normally accompanied with an extensive reprogramming of transcription and translation. In this context, several ribosome-associated proteins are induced under specific conditions in order to re-adapt the function of the ribosome to the new scenario [2]. Some of these proteins are coded by *lrtA* orthologs or paralogs genes. The results presented here demonstrate that the *Synechocystis* LrtA protein is associated to ribosomal particles, indicating that in cyanobacteria, as in other bacterial groups, this protein family has a function in translation.

The *lrtA* gene was originally identified in *Synechococcus* sp. PCC 7002 as a dark-transcribed gene whose mRNA was not detected in the light [3]. In contrast, the *Synechocystis lrtA* gene is expressed under continuous illumination to a significant level. Similar to the *Synechococcus* gene, the level of the *Synechocystis lrtA* transcript is also up-regulated in the dark. As mentioned in the introduction, it has been shown that transcription of *lrtA* in *Synechocystis* takes place mainly by the SigB containing RNA polymerase [22]. This finding was also confirmed by *in vitro* transcription analysis [21]. In darkness, SigB increases and this contributes to the dark-induced *lrtA* gene expression. However, in a *sigB* knockout strain the dark-induced pattern of expression is still observed for the *lrtA* gene [22]. These results clearly indicate that a sigB independent mechanism is partially responsible for the pattern of *lrtA* transcript accumulation. We demonstrate here that *lrtA* transcript from *Synechocystis* is more stable in the dark than in the light and also that the *lrtA* 5’UTR is responsible for this post-transcriptional mechanism. In agreement with our results, light-dependent differences in transcript stability were also reported for the *Synechococcus lrtA* gene [4]. However, we observe that the level of LrtA protein remains constant upon light-dark transitions. Cyanobacterial cells undergo a general translational inhibition upon transfer to the dark [46]. The darkness-dependent increase in the level of *lrtA* transcript could be a mechanism to ensure that the level of LrtA protein remains constant during the dark.

Besides the light/dark regulation of *lrtA* transcript accumulation, the comparative analysis of LrtA protein level in *Synechocystis* cells containing or lacking a leader-less copy of the *lrtA* gene is remarkable. The presence of the leader region has a clear negative effect on the transcript translatability. As mentioned above, extensive secondary structure can be predicted for this region and could be responsible for this effect. As can be observed in the prediction shown in S4 Fig, the ribosome-binding site may be possibly hindered by the secondary structure, which may represent an additional point of control of the *lrtA* expression.

In a classification of cyanobacterial promoters that is based on sigma factors that recognize them, *lrtA* promoter is assigned to type 2 having only the -10 hexamer. Promoters from some nitrogen-regulated genes belong to the same type and contain a motif for the nitrogen control regulator NtcA [20, 21]. We show here that *lrtA* expression is strongly down regulated under nitrogen starvation. Interestingly a putative NtcA binding site centred at position -26.5 upstream of the *lrtA* TSP was found (S7 Fig). This localization of NtcA binding site is characteristic of NtcA-repressed genes [47]. According to our results, NtcA repression of the *lrtA* promoter could be hypothesized. All together these results indicate that the *Synechocystis lrtA* transcript accumulation integrates several environmental signals.

In agreement with the role of LrtA orthologs in other bacteria, association of LrtA protein with ribosomes has been demonstrated in this work. In fact, the six basic amino acids involved in binding between the homologous protein YfiA and the ribosome [19], are conserved in LrtA (Fig 1). However, part of the LrtA protein could be detected in a protein fraction that is not associated with ribosomes. This result indicates that, in agreement with the observations for other members of this protein family, LrtA is a ribosome-associated factor, not a canonical ribosomal protein. Concerning the possible role of LrtA, the results obtained with the *Synechocystis* ∆*lrtA* strain clearly indicate that LrtA stabilizes ribosomal 70S particles under normal growth conditions at all stages of growth. The comparison of ribosomal profiles from the wild type and ∆*lrtA* cells also suggests a drop in the number of ribosomes in the last strain. Nevertheless, with the available data we cannot distinguish between a possible defect in the translation initiation or early blockage of elongation. However no differences could be observed in the growth curves of ∆*lrtA* and WT strains under laboratory conditions.

Drug sensitivity assays carried out with tylosin and erythromycin showed a higher growth of the ∆*lrtA* strain *versus* the wild type in the presence of these antibiotics. Ribosome binding sites of tylosin and erythromycin have been located in approximately the same region of the large ribosomal subunit, within a hydrophobic crevice of the peptide exit tunnel. These antibiotics inhibit protein synthesis by blocking the egress of nascent polypeptides [48, 49]. Members of the LrtA protein family (YfiA, HPF) have ribosome binding sites that overlap with those of the mRNA, transfer RNA and initiation factors [7]. This localization in the intersubunit entrance-exit channel is relatively close to the macrolides binding site, which may explain why the presence or absence of LrtA modifies the effect of tylosin and erythromycin in *Synechocystis* cells.

One of the best-characterized proteins of the LrtA family is the *E*. *coli* YfiA [50]. *In vitro* studies demonstrated that this protein inhibits translation and is present in ribosomes of cells that reached stationary phase or are subjected to a temperature downshift [51]. Structural work established that YfiA binds to the 30S subunit and probably stabilizes a state of this subunit with high affinity for the 50S subunit, thereby preventing subunit dissociation. The role attributed to this protein was to modulate ribosome activity as a function of cell stress and transiently repress protein synthesis, specifically during cold adaptation [19]. The other *E*. *coli* protein of the family, YhbH or HPF, is also related to the down-regulation of protein synthesis, upon entry into stationary phase [7]. In contrast, LrtA protein from *Synechocystis* is present in ribosomes isolated from exponentially growing cells and moreover it is down-regulated in stationary phase or nitrogen-starved cells. Thus, LrtA function does not seem to be related to the translation during periods of limited growth. Rather, the results presented here indicate a role of LrtA in post-stress survival, specifically when growth restarts after long starvation periods. As mentioned in the introduction, LrtA belongs to the long HPF type. Proteins similar in length, sequence and domain structure are present in other bacterial groups [12]. Similar to the case of *Synechocystis*, these bacterial species have no orthologues of *E*. *coli rmf* and *hpf*. Although *in vivo* studies of these proteins are limited, a detailed analysis of *Lactococcus lactis* YfiA, a long HPF type protein, has been recently published [52]. This study demonstrates that deletion of the *yfiA* gene has no effect on the growth rate but diminishes the survival of *Lactococcus lactis* under energy-starving conditions. Parallel to what we observed with LrtA in cyanobacteria, the growth of *Lactococcus lactis* ∆*yfiA* was reduced compared to wild type upon longer starvation and the lag times before re-growth increased more than that of wild type after prolonged starvation. These results suggest that the *in vivo* effect of lacking such proteins is quite similar.

Based on our results, we cannot consider that LrtA is a stress protein; rather our working hypothesis is that LrtA must have a function under normal growth conditions, but may not be easily observable as a difference in the mutant growth curve under laboratory conditions. In fact it has been described that long type HPF proteins from other bacteria are continually expressed throughout all growth phases [12]. To explain the positive effect of LrtA in restoring growth after stress, a role in re-association of ribosomal subunits can be hypothesized in view of our observation on the amount of 70S ribosomal particles in WT and mutant strains (Fig 7). Localization of *Synechocystis* LrtA in the ribosome must be the same that has been described for other proteins of the family (pY or PSRP-1) namely, overlapping mRNA and tRNAs positions [18, 19]. Consistently with studies of these proteins [8, 11], LrtA could be released from the 70S ribosome by translation factors such as RRF and EF-G, leading to subunit dissociation and translation initiation mediated by IF3 and other initiation factors. We speculate that LrtA-mediated stabilization of 70S ribosomes may have a function in the physiology of *Synechocsytis* and not just as a “storing” factor involved in sequestering a fraction of the ribosomes as idle 70S monomers, when a lower number of translating ribosomes is required by the cell, as have been proposed for pY protein.

The results we have obtained in experiments with sorbitol are intriguing (Fig 9), since strains lacking LrtA are able to grow much faster than the wild-type in the presence of 0.5 M sorbitol. It has been reported that incubation of *Synechocystis* cells in medium supplemented with 0.5 M sorbitol decreases the cytoplasmic volume to 30% of the original value within 10 min and then remained at this level [44]. Consistent with our hypothesis about LrtA function, it is possible that the absence of this protein favours the normal association/dissociation cycle of ribosomal subunits in the translation process with the hyperosmotic pressure provoked by sorbitol. This would explain the growth results obtained with strains with different LrtA protein level, as well as the down-regulation in LrtA abundance observed in wild-type cells after sorbitol treatment.

It has been described that heat shock induces ribosome subunit dissociation and translating ribosome can dissociate erroneously [2]. It is interesting the observation that *lrtA* transcript abundance is temperature-dependent in *Synechocystis* (S8 Fig). The transcript is up-regulated at high temperature, probably because of the increase of SigB sigma factor in these conditions [20]. This result also supports a role of LrtA on the stabilization of the 70S particles.

Although several aspects of the LrtA function are not yet known, this work is, to our knowledge, the first study that addresses the role of this family of proteins in cyanobacteria.

## Supporting Information

S1 Fig Scheme of modified *loci* in the constructed strains. *A*, Schematic representation of the *lrtA* genomic region in the WT and in the ∆*lrtA* mutant strains. ∆*lrtA*(+) and ∆*lrtA*(-) refer to the orientation of the antibiotic resistance cassette in the same or opposite direction respect to the *lrtA* gene, respectively. *B*, Southern blot analysis of *Synechocystis* WT and ∆*lrtA* mutant. Genomic DNA was digested with *Hinc*II and *Hae*II, and hybridized using the DNA fragment indicated in panel *A* as a probe. *C*, Northern blot analysis of *Synechocystis* WT and the ∆*lrtA* mutant. Total RNA was isolated from 15 min dark-incubated *Synechocystis* cells and *lrtA* transcript was detected by Northern blotting. Then the filter was stripped and rehybridized with a *rnpB* probe as loading control. *D*, *Synechocystis* WT and ∆*lrtA* mutant total extract proteins were separated by SDS-PAGE and subjected to Western blotting using anti-LrtA antibodies. *E*, Schematic representation of the *nrsBACD locus* in the *Synechocystis* LrtAC strain. *F*, Schematic representation of the *nrsBACD locus* in the *Synechocystis* LrtAS strains.
(TIF)

S2 Fig Darkness-dependent regulation of *lrtA* mRNA. Total RNA was isolated from early-log-phase *Synechocystis* cells growing under normal illumination conditions (Light, time 0) or after being subjected to darkness for 15, 30, 60, 240 or 480 min. 7 μg of total RNA was loaded per lane. Level of *lrtA* mRNA was determined by Northern blotting. The filter was stripped and re-hybridized with a *rnpB* gene probe as loading control. The values represented in the histogram are relative to that of cells after 15 min in darkness (100%).
(TIF)

S3 Fig Primer extension analysis. Oligonucleotide used for primer extension analysis of the *lrtA* transcript was lrtAR4 (S1 Table), complementary to positions—76 to -95 relative to the translation start of *lrtA*. Primer extension assays were carried out with RNA isolated from cells growing under normal illumination conditions (Light) or subjected to darkness for 15 min. End-labelled 20-pb DNA ladder (Bio-Rad) was used as a marker.
(TIF)

S4 Fig *lrtA* leader region. Predicted secondary structure o the 5' UTR of *lrtA* (from -312 to +10 with respect to translational start) according to Mfold [53]. The transcriptional start [42], Shine-Dalgarno region (SD) and translational start (boxed) are indicated.
(TIF)

S5 Fig Effect of different antibiotics on the growth of *Synechocystis* wild type and ∆*lrtA* mutant. Ten-fold serial dilutions of each *Synechocystis* culture were spotted on BG11C medium plates (control) or in BG11C supplemented with the indicated concentration of antibiotic and photographed after 10 days of growth. ∆*lrtA*(+) refer to the orientation of the antibiotic resistance cassette in the same direction respect to the *lrtA* gene.
(TIF)

S6 Fig Growth of *Synechocystis* strains with different amounts of LrtA, in refreshing conditions after nitrogen starvation. Early-log-phase *Synechocystis* cells of the wild-type and mutant strains growing photoautotrophically (50 μmol photons m^-2^ s^-1^, 1% (v/v) CO_2_ in air) were centrifuged, resuspended in BG11_0_C medium (lacking any nitrogen source) and incubated under normal illumination conditions for one week. These cells were used to inoculate fresh BG11C medium at 0.1 OD_730_ (day 1). Growth was followed for 5 days. The data represent average values of three independent experiments. ∆*lrtA*(+) and ∆*lrtA*(-) refer to the orientation of the antibiotic resistance cassette in the same or opposite direction respect to the *lrtA* gene, respectively.
(TIF)

S7 Fig Alignment of different NtcA-repressed promoter sequences. NtcA binding sites are chaded in gray, -10 regions are boxed and the transcriptional start is underlined. The consensus sequence of the NtcA-activated promoter (centered at -41.5) is also shown. *gifA* 7120 (*gifA* gene from *Anabaena* sp. PCC 7120 [54]), *gifA* 6803 (*gifA* gene from *Synechocystis* sp. PCC 6803 [47]), *gifB* 6803 (*gifB* gene from *Synechocystis* sp. PCC 6803 [47]), *rbcL* 7120 (*rbcL* gene from *Anabaena* sp. PCC 7120 [55]).
(TIF)

S8 Fig Temperature-dependent regulation of *lrtA* mRNA. Early-log-phase *Synechocystis* cells growing under normal conditions at 30°C (time 0) were transferred to different temperatures and total RNA was isolated at the indicated times. 7 μg of total RNA was loaded per lane. Level of *lrtA* mRNA was determined by Northern blotting. The filter was stripped and re-hybridized with a *rnpB* gene probe as loading control. The values represented in the histogram are relative to that of control cells after 15 min at 30°C (100%).
(TIF)

S1 Table Oligonucleotides used in this study. (DOCX)
