# Supplementary figures and images for: The Cyanobacterial Ribosomal-Associated Protein LrtA Is Involved in Post-Stress Survival in Synechocystis sp. PCC 6803
Source: PLoS One. 2016 Jul 21;11(7):e0159346. doi: 10.1371/journal.pone.0159346 (PMC4956104; doi:10.1371/journal.pone.0159346)

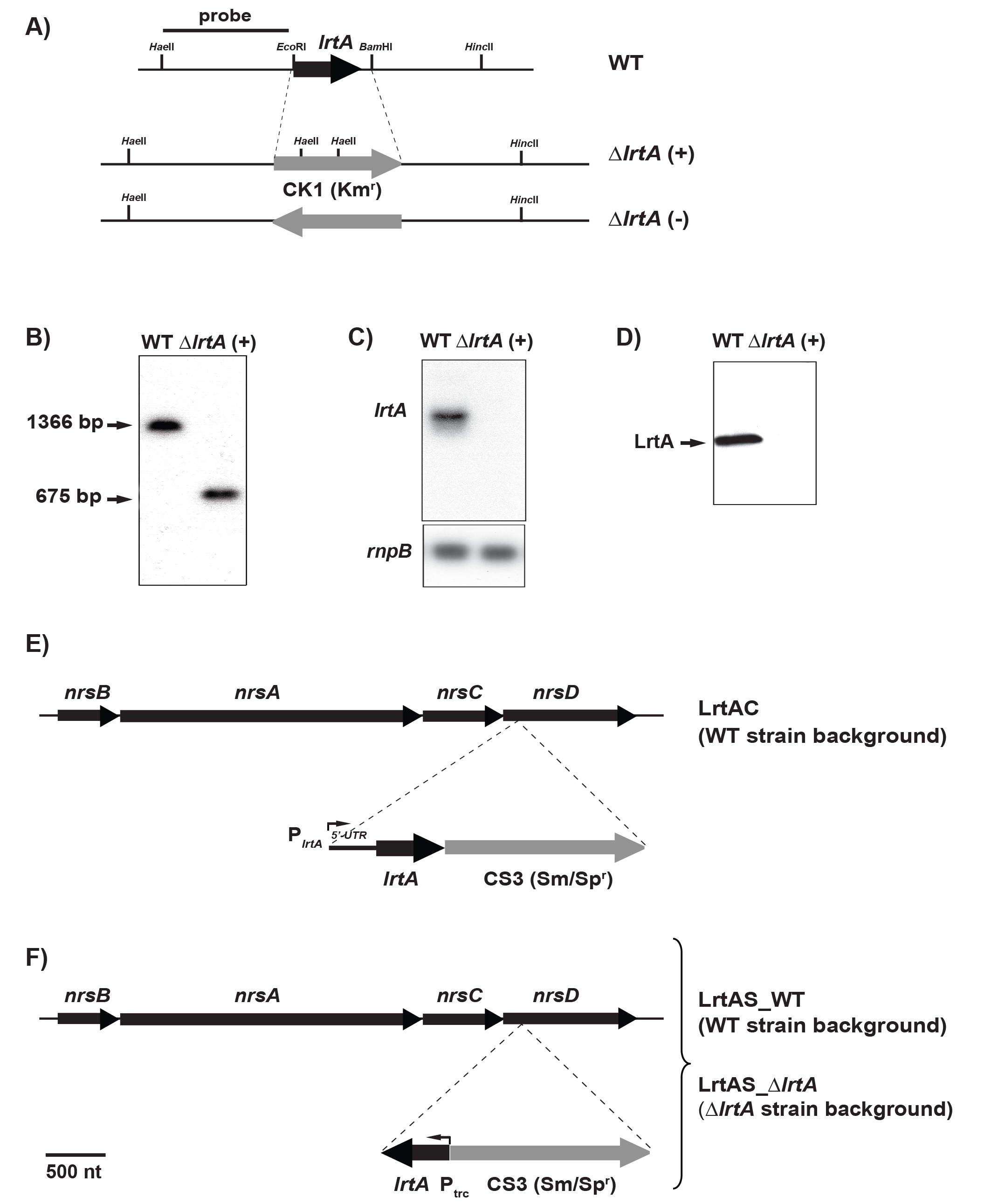

Supplement: S1 Fig — A, Schematic representation of the lrtA genomic region in the WT and in the ∆lrtA mutant strains. ∆lrtA(+) and ∆lrtA(-) refer to the orientation of the antibiotic resistance cassette in the same or opposite direction respect to the lrtA gene, respectively. B, Southern blot analysis of Synechocystis WT and ∆lrtA mutant. Genomic DNA was digested with HincII and HaeII, and hybridized using the DNA fragment indicated in panel A as a probe. C, Northern blot analysis of Synechocystis WT and the ∆lrtA mutant. Total RNA was isolated from 15 min dark-incubated Synechocystis cells and lrtA transcript was detected by Northern blotting. Then the filter was stripped and rehybridized with a rnpB probe as loading control. D, Synechocystis WT and ∆lrtA mutant total extract proteins were separated by SDS-PAGE and subjected to Western blotting using anti-LrtA antibodies. E, Schematic representation of the nrsBACD locus in the Synechocystis LrtAC strain. F, Schematic representation of the nrsBACD locus in the Synechocystis LrtAS strains. (TIF) [file pone.0159346.s001.tif]

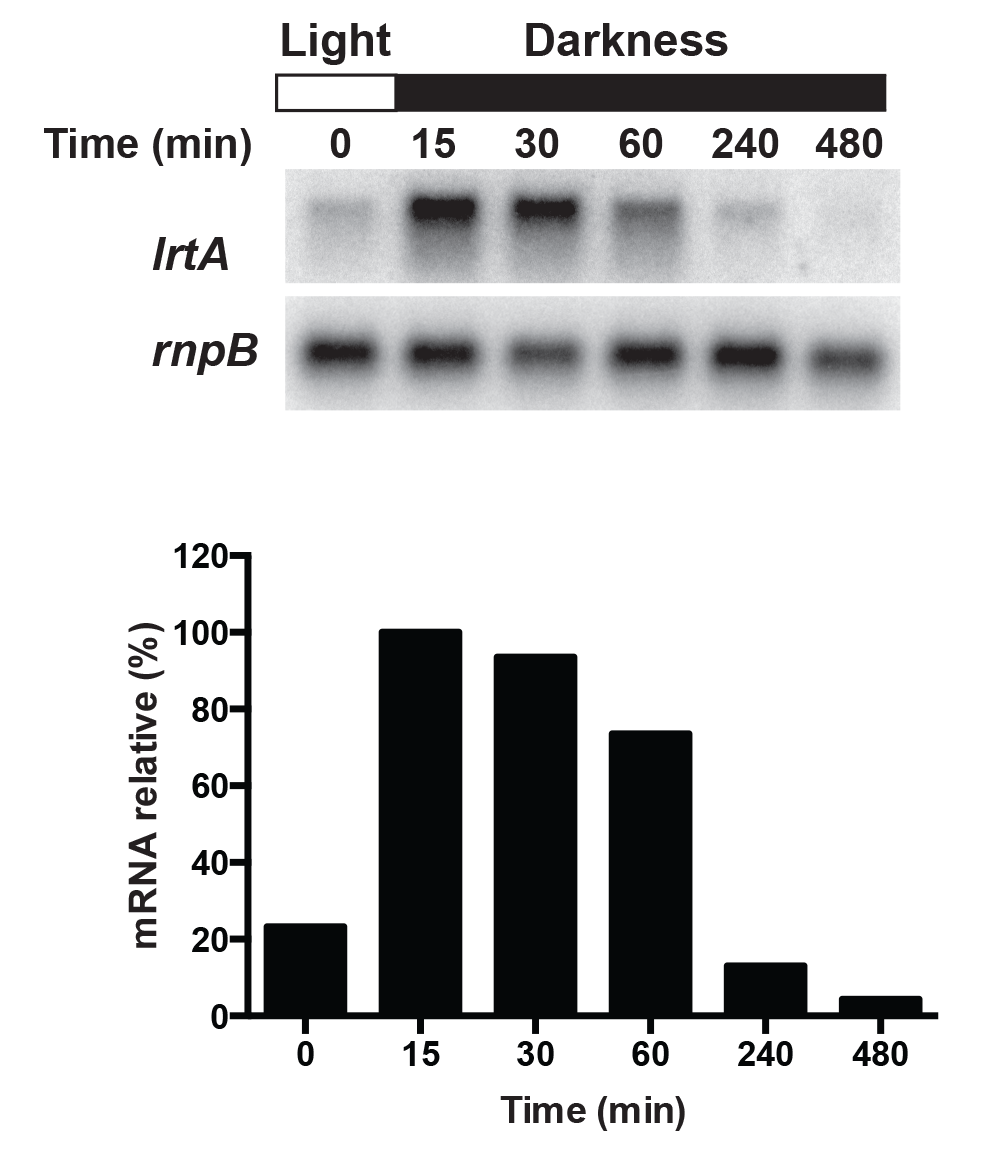

Supplement: S2 Fig — Total RNA was isolated from early-log-phase Synechocystis cells growing under normal illumination conditions (Light, time 0) or after being subjected to darkness for 15, 30, 60, 240 or 480 min. 7 μg of total RNA was loaded per lane. Level of lrtA mRNA was determined by Northern blotting. The filter was stripped and re-hybridized with a rnpB gene probe as loading control. The values represented in the histogram are relative to that of cells after 15 min in darkness (100%). (TIF) [file pone.0159346.s002.tif]

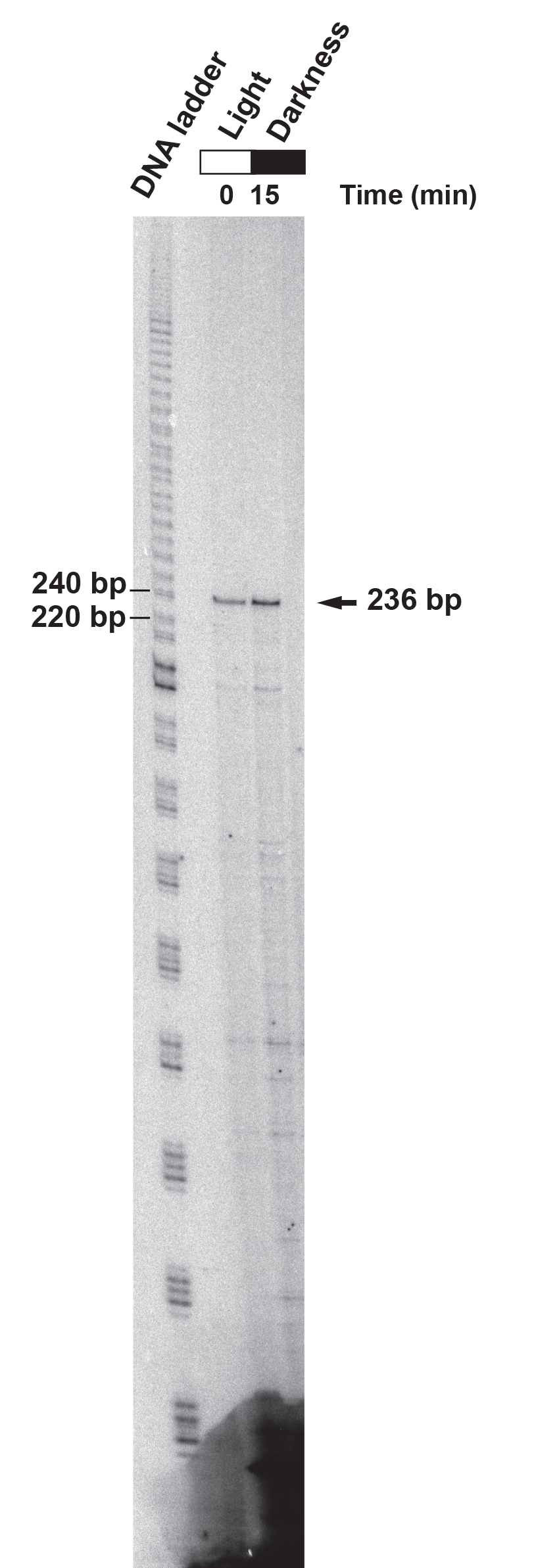

Supplement: S3 Fig — Oligonucleotide used for primer extension analysis of the lrtA transcript was lrtAR4 (S1 Table), complementary to positions—76 to -95 relative to the translation start of lrtA. Primer extension assays were carried out with RNA isolated from cells growing under normal illumination conditions (Light) or subjected to darkness for 15 min. End-labelled 20-pb DNA ladder (Bio-Rad) was used as a marker. (TIF) [file pone.0159346.s003.tif]

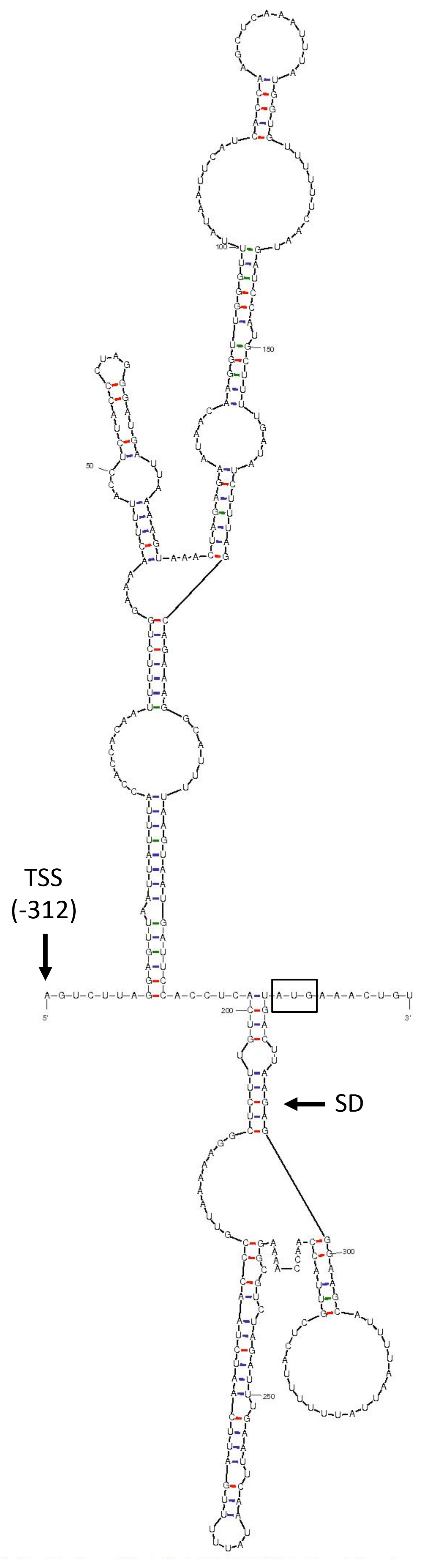

Supplement: S4 Fig — Predicted secondary structure o the 5' UTR of lrtA (from -312 to +10 with respect to translational start) according to Mfold [53]. The transcriptional start [42], Shine-Dalgarno region (SD) and translational start (boxed) are indicated. (TIF) [file pone.0159346.s004.tif]

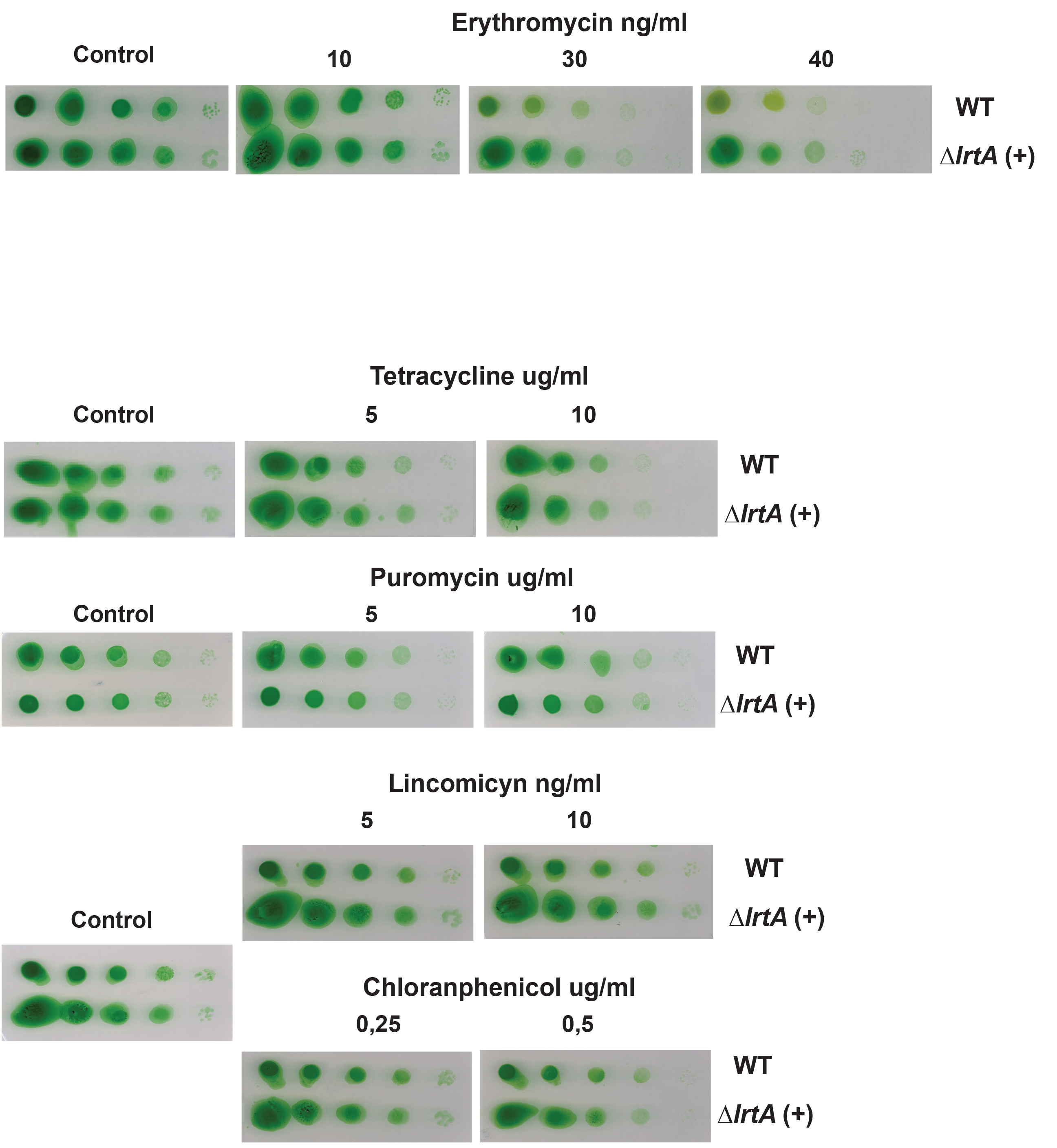

Supplement: S5 Fig — Ten-fold serial dilutions of each Synechocystis culture were spotted on BG11C medium plates (control) or in BG11C supplemented with the indicated concentration of antibiotic and photographed after 10 days of growth. ∆lrtA(+) refer to the orientation of the antibiotic resistance cassette in the same direction respect to the lrtA gene. (TIF) [file pone.0159346.s005.tif]

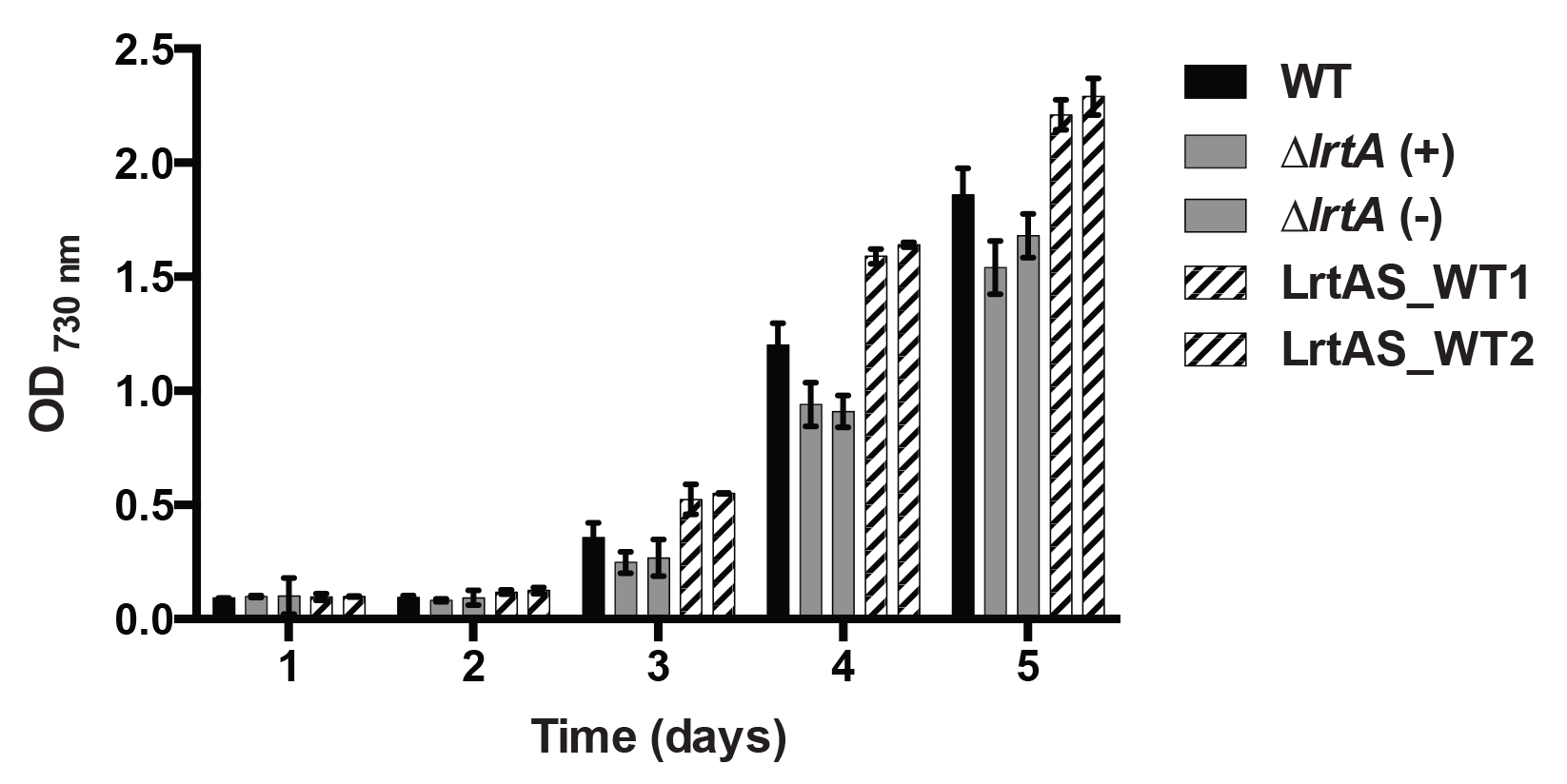

Supplement: S6 Fig — Early-log-phase Synechocystis cells of the wild-type and mutant strains growing photoautotrophically (50 μmol photons m-2 s-1, 1% (v/v) CO2 in air) were centrifuged, resuspended in BG110C medium (lacking any nitrogen source) and incubated under normal illumination conditions for one week. These cells were used to inoculate fresh BG11C medium at 0.1 OD730 (day 1). Growth was followed for 5 days. The data represent average values of three independent experiments. ∆lrtA(+) and ∆lrtA(-) refer to the orientation of the antibiotic resistance cassette in the same or opposite direction respect to the lrtA gene, respectively. (TIF) [file pone.0159346.s006.tif]

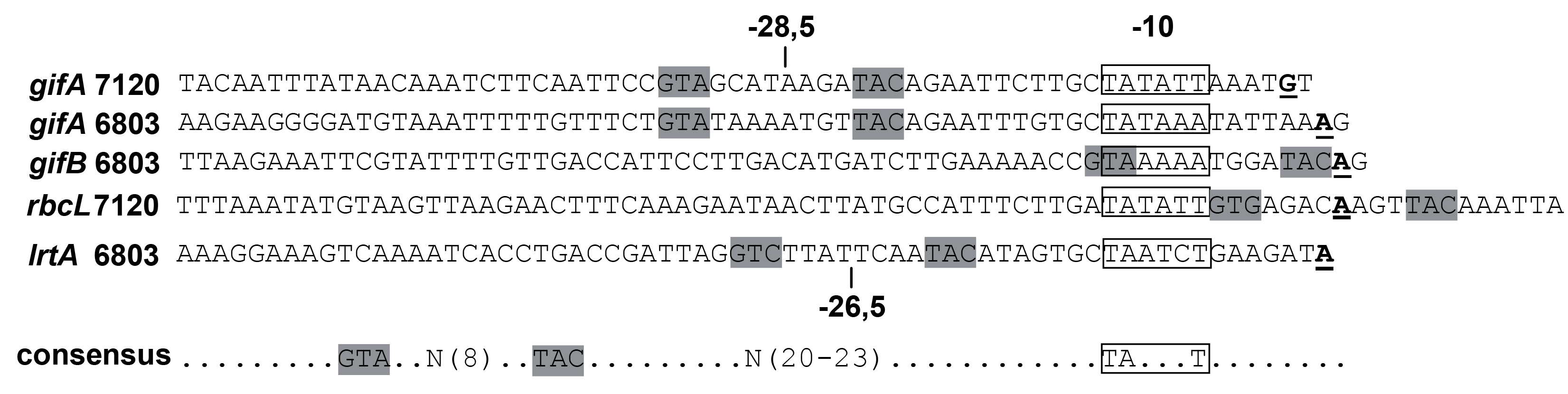

Supplement: S7 Fig — NtcA binding sites are chaded in gray, -10 regions are boxed and the transcriptional start is underlined. The consensus sequence of the NtcA-activated promoter (centered at -41.5) is also shown. gifA 7120 (gifA gene from Anabaena sp. PCC 7120 [54]), gifA 6803 (gifA gene from Synechocystis sp. PCC 6803 [47]), gifB 6803 (gifB gene from Synechocystis sp. PCC 6803 [47]), rbcL 7120 (rbcL gene from Anabaena sp. PCC 7120 [55]). (TIF) [file pone.0159346.s007.tif]

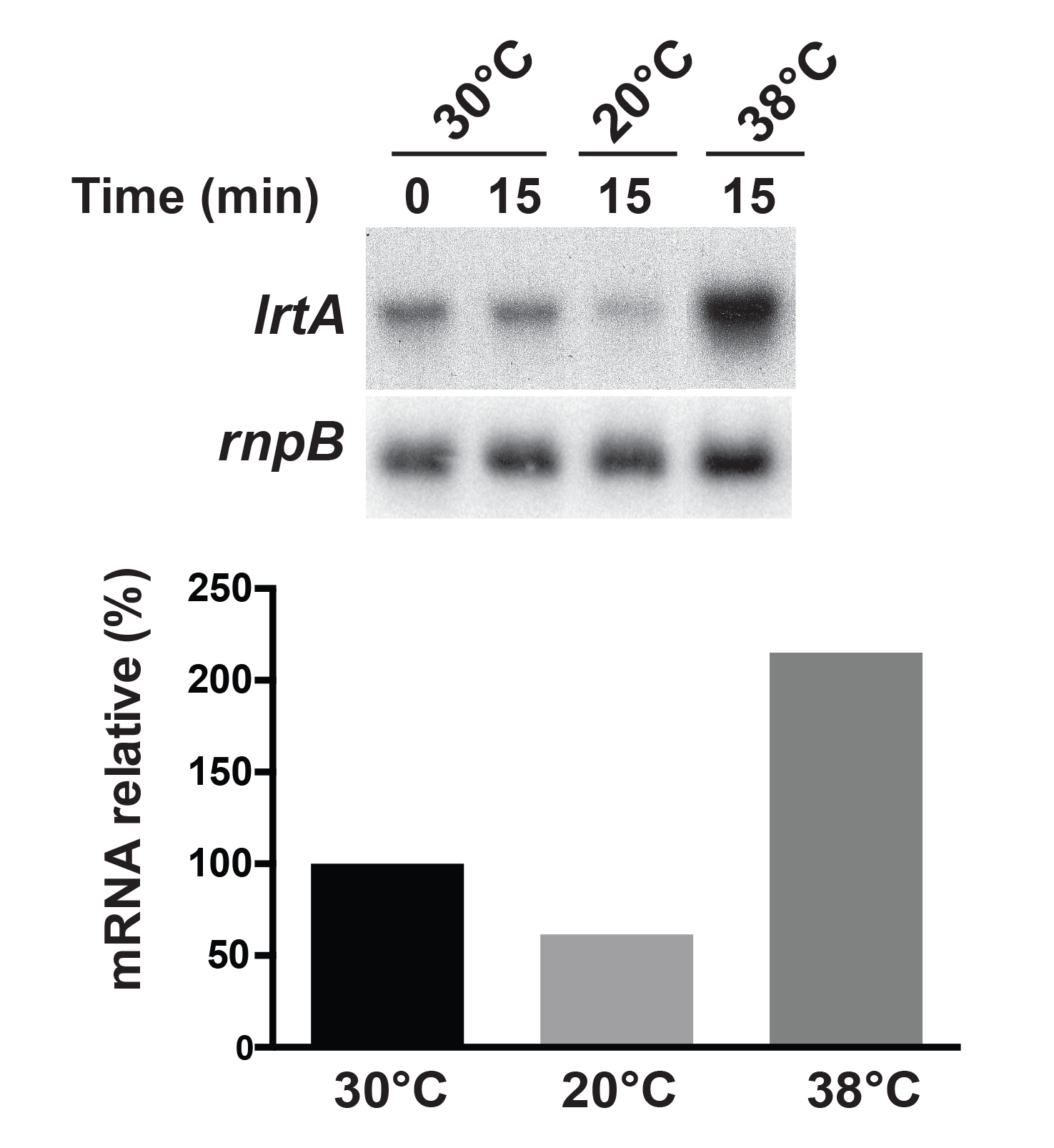

Supplement: S8 Fig — Early-log-phase Synechocystis cells growing under normal conditions at 30°C (time 0) were transferred to different temperatures and total RNA was isolated at the indicated times. 7 μg of total RNA was loaded per lane. Level of lrtA mRNA was determined by Northern blotting. The filter was stripped and re-hybridized with a rnpB gene probe as loading control. The values represented in the histogram are relative to that of control cells after 15 min at 30°C (100%). (TIF) [file pone.0159346.s008.tif]
