# Supplementary material for: The Cyanobacterial Ribosomal-Associated Protein LrtA Is Involved in Post-Stress Survival in Synechocystis sp. PCC 6803
Source: PLoS One. 2016 Jul 21;11(7):e0159346. doi: 10.1371/journal.pone.0159346 (PMC4956104; doi:10.1371/journal.pone.0159346)
Supplement: S1 Table — (DOCX) [file pone.0159346.s009.docx]

| **Oligonucleotide** | **Sequence** |
| --- | --- |
| lrtA1 | 5’CCAACAACATTAGTCCGTCC3’ |
| lrtA2 | 5’CAGGGCTACAACGACACCATTC3’ |
| lrtASmaI | 5’GTGTTTATTCCCCCGGGTTAGCTTTGC3’ |
| lrtAKpnI | 5’CCATGGCGGTACCAAGGAAACGGAATC3’ |
| lrtABspHI | 5’GACTCATGAAACTGTTAATTCAGGGCAATAATATCACAGTTACTGAAGCGATTCACGATTACGTG3’ |
| lrtASmaI.2 | 5’GATCCCCGGGCTAGCTGGCTTGGTGGGGTTGAATAACG3’ |
| lrtAR4 | 5’AAAACTAAGTTAGATTGGGC3’ |

**S1 Table. Oligonucleotides used in this study**
